# Supplementary material for: Smart textile lighting/display system with multifunctional fibre devices for large scale smart home and IoT applications
Source: Nat Commun. 2022 Feb 10;13:814. doi: 10.1038/s41467-022-28459-6 (PMC8831553; doi:10.1038/s41467-022-28459-6)
Supplement: Supplementary file 1 — Supplementary Information [file 41467_2022_28459_MOESM1_ESM.pdf]

Supplementary Information for

Smart textile lighting/display system with multifunctional fibre devices for large scale smart home and IoT applications

Hyung Woo Choi[1,†], Dong-Wook Shin[1,†], Jiajie Yang[1,†], Sanghyo Lee[1,†], Cátia Figueiredo[2], Stefano Sinopoli[3], Kay Ullrich[4], Petar Jovančić[5], Alessio Marrani[6], Roberto Momentè[7], João Gomes[8], Rita Branquinho[2], Umberto Emanuele[3], Sang Yun Bang[1], Hanleem Lee[1], Sung-Min Jung[1], Soo Deok Han[1], Shijie Zhan[1], William Harden-Chaters[1], Yo-Han Suh[1], Xiang-Bing Fan[1], Tae Hoon Lee[1], Mohamed Chowdhury[1], Youngjin Choi[1], Salvatore Nicotera[3], Andrea Torchia[3], Francesc Mañosa Moncunill[5], Virginia Garcia Candel[5], Nelson Durães[8], Kiseok Chang[9], Sunghee Cho[9], Chul-Hong Kim[9], Marcel Lucassen[10], Ahmed Nejim[11], David Jiménez[12], Martijn Springer[13], Young-Woo Lee[14,15], SeungNam Cha[14,16], Jung Inn Sohn[14,17], Rui Igreja[2], Kyungmin Song[18], Pedro Barquinha[2], Rodrigo Martins[2], Gehan Amaratunga[1], Luigi G. Occhipinti[1,*], Manish Chhowalla[20,*], Jong Min Kim[1,*]

Correspondence to: lgo23@cam.ac.uk, mc209@cam.ac.uk, jmk71@cam.ac.uk

This PDF file includes:

Supplementary Notes 1 to 9
Supplementary References
Supplementary Figs. 1 to 25
Supplementary Tables 1 to 5

Supplementary Note 1. Fibre devices (F-devices)

All F-devices fitted to weaving/interconnection process are specifically designed and optimised for our textile system. All fibre devices show the aspect ratio over 30 (length-to-width with a few millimetre-scale) to incorporate them into a textile replacing warp (vertical)/weft (horizontal) cotton fibres; F-LED (extraction/increment of directional light), F-photodetector (maximising incoming photon collection), and fibre transistor (easiness of three terminal interconnection with conductive fibre) are fabricated on planar fibre substrate, and the other F-devices (F-RF, F-touch, F-temperature, and F-energy) are fabricated on cylinder fibres.

Supplementary Note 2. Mechanical stability of conductive fiber

Conductive fibres (Ag-PA) exhibit outstanding mechanical and electrical stability against repeated external deformations (bending radius ≥ 1 mm). To further explore the mechanical limit of our conductive fibre, a stretching test was performed (Supplementary Fig. 14a: strain 10%, Supplementary Fig. 14b: strain 20%)[1,2]. Under the uni-axial strain condition, Ag-PA fibers showed unchanged current (at 10^{-4} A), which indicated Ag layer holds conductive pathway under a given strain condition. It was interesting that cyclic stretching test revealed stress concentration in the early stage of deformation. A continuous decrease in stress showed accumulated plastic strain behavior after the first stretching period [3]. We found that the initial stretching period critically influenced plastic deformation of conductive fiber based on the stress mapping (Supplementary Fig. 14 color maps). Then, less stress field was applied to core fiber (PA) resulting in a continuous decrease in stress over time. As any smart textile shape-alteration (Fig. 1) does not exceed 20% of strain conditions, our conductive fiber shows compatibility with rolling, bending, and folding conditions. However, it is worth to mark that stretching limit of Ag-PA fiber is 35% when the percolative network of Ag layer is broken (Supplementary Fig. 15)

Supplementary Note 3. F-RF antenna operating principle

AC signal (from RF transmitter) applied to the antenna passes through a bridge rectifier where the DC voltage is obtained and optimized for signal detection at the controller unit (Supplementary Fig. 16a). The strength of the induced voltage was used to measure output characteristics without making physical contact between the RF transmitter and the antenna. In the F-RF without a bridge rectifier, AC voltage and output power are directly measured from the antenna as a function of distance between the RF power transmitter and F-RF (Supplementary Fig. 16b,c). To classify signal intensity visualizing on the textile system, the DC voltage is measured from the bridge rectifier that is connected to a load. The output voltage varies in the range of approximately 1 V to 7 V as a function of loads (Supplementary Fig. 16d). With a voltage suppressing resistor (560 Ω), the maximum DC voltage at 2.5 V ($Distance_{transmitter}$ = 0 cm) was obtained and the maximum current recorded 3.56 mA leading total power consumption of 8.9 mW (Supplementary Fig. 16d,e). Output voltage was found to gradually decrease from 2.5 V down to 0.2 V with distance between RF power generator from the embroidered antenna (0 to 60 mm in distance for near field activation) (Supplementary Fig. 16f). This output voltage was to indicate the number of bars (six-segmentation levels) on the textile display.

Supplementary Note 4. F-photodetector operating principle and application

In the woven F-photodetector, as current range (10^{-7} A) was not suitable for reading signals at the controller, we converted the current reading to voltage readings by connecting a load and a capacitor (Supplementary Fig. 17a–c). The optimized output voltage of F-photodetector at the peak (UV on) was measured ~ 2 V (Supplementary Fig. 17d). At the controller side, the on-state and off-state voltage were set to 1.5 V and 0.5 V, respectively (Supplementary Fig.

17e). In this manner, UV responsive photodetector is accomplished without measuring tools such as Keithley semiconductor analyzer (Supplementary Fig. 17f). Compared with state-of-the-art flexible ZnO-based photodetector[4-15], our F-photodetector exhibits average I_{light}/I_{dark} ratio (10^2), low operating voltage (10 V), and rise time (3 sec) (Supplementary Fig. 6).

However, the novelty of F-photodetector lies in real-life application. The smart textile over large scale can be installed covering in any wall or window, *e.g.* a smart curtain. When F-photodetector is facing outwards, UV sensitive active material continuously read UV ratings. In this regard, we show sunlight responsivity of F-photodetector (Supplementary Fig. 18a,b). The sunlight intensity (solar simulator, 100 mW/cm^2, AM1.5G, 100%) was controlled by UV filters (from 15% up to 100%, 5 sets) that were located on top of F-photodetector. At 15 V applying to electrodes, the photocurrent of F-photodetector ranges from 4.38×10^{-8} A to 3.41×10^{-7} A that enables to advise users whether the smart textile system is rolled down (UV 100%) or up (below UV 50%) (Supplementary Fig. 18c). From I_{light}-V measurements, we can extract Schottky barrier height where I_o can be obtained by extrapolating I-V curve at a given applied voltage (10 V)[16,17].

$$I_0 = AA^*T^2 \exp\left(-\frac{q\Phi_B}{kT}\right) \tag{1}$$

Where A is the contact area, A^* is the Richardson constant $(4\pi qmk^2/h^3)$, k is Boltzmann constant, T is the absolute temperature, q is the element charge, and Φ_B is the Schottky barrier height. Extracted Schottky barrier height was 0.532 eV that is built on ZnO-Al metal-semiconductor junction at room temperature.

Supplementary Note 5. F-touch sensor operating principle and application

The interconnection of each conductive thread (diameter, approximately 100-200 μm) comprising 30 sensor points in the F-touch to a controller was made by clamping each thread into a pin-connector to form an electrical circuitry (Supplementary Fig. 19). To implement practical use, we coded internet of things (IoT) terms into each F-sensor element (Supplementary Fig. 20, Table 5). All the execution codes are related to current state-of-the-art smart home gadgets.

In addition to native F-touch performance, the critical assessment of the speed of touch sensor and reliability of each sensor element were performed prior to the F-touch sensor integration (Fig. 3c). Owing to its surface responsivity of conductive fibers, a critical resistance variation is required to read it as an input signal as small as 2% variation. Based on measured data (data acquisition interval = 6 ms), input touch was read as fast as 18 ms reflecting fast response speed compared to reported touch sensors[18-20]. Reliability test was performed in conjunction with cyclic touch stability test. As a nature of resistive touch sensor, conductive fiber is subject to experience surface weariness until it misreads ΔR $(R_{touch} - R_{release})$ values. When on-state voltage was set to $\Delta R/R_{max} \times 100 = 50\%$ (0.5 V) for activating the smart textile system, 3000 continuous touch attempts were carried out to identify weariness. Voltage readings for on-states (higher than threshold = 50%, the average of all the readings: 0.775 V ($\Delta R/R_{max}$, relative standard deviation of 4.53%), SNR>70 dB) were 100% as same as off-state records (lower than 0.5 V). In this regard, we have confirmed that our conductive fiber is able to hold several thousand push cases without surface damage.

Supplementary Note 6. F-temperature sensor operating principle and reliability over time

F-temperature sensors were weft-replaced in the smart textile system, followed by interconnecting to a controller (Supplementary Fig. 21a). It is used to obtain the resistance (or current) values from which a real-time temperature is calculated and displayed (Supplementary Fig. 21b,c).

As known to be a p-type semiconductor showing a negative temperature coefficient, the resistance of Cu_2O inversely correlates with the temperature. To evaluate fiber-type Cu/Cu_2O resistance dependency with respect to temperature, twisted Cu/Cu_2O fibers were placed on a hot plate[21].

$$R = R_{ref}\left[1 + \alpha\left(T - T_{ref}\right)\right] \quad (2)$$

Where R is resistance at measured temperature, R_{ref} is resistance at the reference temperature (25 °C), α is the temperature coefficient of resistance for specified material (Cu_2O), and T_{ref} is reference temperature.

$$\Delta R/R_{ref} = \alpha\Delta T \quad (3)$$

$$\alpha = -\frac{\beta}{T^2} \quad (4)$$

$$\beta = \ln\left(\frac{R_{T1}}{R_{T2}}\right)/(\frac{1}{T_1} - \frac{1}{T_2}) \quad (5)$$

Where β is a temperature constant (K). At 5 to 70 °C, β = 1283±5 K was obtained from measured resistance variation (Supplementary Fig. 9a). The classification of temperature along with resistance change is achieved in the range from 5 °C up to 70 °C (Supplementary Fig. 9b).

The estimated sensitivity of F-temperature sensor was obtained by maintaining hot-plate temperature at 25 °C for several minutes [22]. Measured temperature coefficient of resistance of F-temperature sensor at 25 °C was 1.44%/K where the current fluctuation is less than 10%. Compared to the state-of-the-art fiber temperature sensor[22] with novel material and processing method, our F-temperature sensor shows comparable sensitivity of 0.1 °C (Supplementary Fig. 9c).

Thermal activation energy was extracted from fast temperature increase from 5 °C to 60 °C. Thermal activation is a function of temperature and material property ($R \propto \exp\left(-E_a/K_B T\right)$, where K_B is Boltzmann constant, T is the temperature, and E_a is the activation energy). The measurements show $E_a = 0.266\ eV$ which reveals that the activation energy of Cu_2O correlates to copper vacancy (Supplementary Fig. 9d)[23].

Long-term stability is the key parameter for sensor material where resistance of oxide material varies its conductance as a function of charge carriers. In this regard, copper oxide has been used for gas sensing purposes[24-26]. Even with an encapsulating layer (epoxy), we found non-ideal oxygen blockage over a year. Resistance of F-temperature sensor increased as shown in Supplementary Fig. 9e. However, the variation tendency of current was kept. To compensate measured current variation to display as a temperature, programming code was modified.

Supplementary Note 7. F-biosensor module operating principle and reliability over time

The F-biosensor module comprises fiber-based transistor, a resistor, and electrocardiogram (ECG) pad, based on an amplifier in a common-source configuration, as shown in Supplementary Fig. 10. The transfer characteristics of fiber IGZO transistor shows the threshold voltage (V_{TH}) = 0.9 V, ON/OFF ratio = 10^8, and saturation mobility (μ_{sat}) = 8 $cm^2/V \cdot s$ (Supplementary Fig. 11a,b). In this configuration small change in gate voltage cause large changes in output voltage with a consequence of high amplifier gain ($A_v = \partial V_{out}/\partial V_{in}$) with a load resistor. To fabricate a fiber transistor which replaces a weft thread as well as interconnecting each electrode with conductive fiber in the smart textile system, we made a row of transistor instead of making an amplifier circuit as a proof of concept. As shown in

Supplementary Fig. 11c, amplifier gain was measured to be 28 V/V at the peak. Measured output power (P_{out}) was 350 nW (at the peak) which indicates the total power consumption is low (Supplementary Fig. 11d). The route to increase amplification gain, subthreshold regime is useful for higher peak gain while achieving lower operation power[27] after a transistor evinces mechanical stability.

To evaluate the mechanical flexibility of IGZO transistor, it was attached to cylindrical objects with a specific radius from 5 mm to 13 mm (Supplementary Fig. 11e). The bending strain was applied parallel to the channel of the transistor. As demonstrated in Fig. 3e, applying bending strain has no influence on transfer characteristics until the bending radius reaches 5 mm. Demonstrated bending of smart textile does not show the extreme condition of 5 mm of bending radius, 7 mm bending radius is practical. Compared to state-of-the-art studies, our transistor exhibits small subthreshold swing and high bending stability (Supplementary Fig. 11f)[28-45]. In addition to mechanical stability, we have examined material stability by measuring transfer characteristics of the transistor. The encapsulation layer (parylene-C) on the channel area strongly prevented environmental effect that mobility and threshold voltage variation were $\Delta\mu_{sat}$ is less than 0.45 $cm^2/V{\cdot}s$ and ΔV_{TH} is 0.35 V over a year (Supplementary Fig. 11a,b).

Supplementary Note 8. F-energy storage stability

In order to increase total capacitance of the fiber supercapacitor, we added conductive fiber (Ag-PA) into carbon fiber bundle (schematically illustrated in Supplementary Fig. 12b). Average total capacitance of 30 F-supercapacitors with conductive fiber showed 3-fold increase (Supplementary Fig. 12c). Typical capacitive cyclic voltammetry (CV) curves and variation of capacitance (C/C_{100mV}) at scan rates of 10 mV/s to 100 mV/s is shown in Supplementary Fig. 12d,e. To validate either higher voltage output or larger capacitance for longer textile display operation, we connected 60-cm long F-supercapacitors in series or parallel manner that shows the maximum output voltage and total capacitance of 5 V (5.4 mF) and 22 mF (2 V), respectively (Supplementary Fig. 12f). In comparison with state-of-the-art studies in fiber-based supercapacitors, our F-supercapacitor with conductive fiber shows high specific energy and specific power (Supplementary Fig. S12g)[46-56]. Capacitive stability under mechanical deformation of F-energy storage was tested for demonstrating the capability of mechanical alteration of smart textile system. As shown in Fig 3f and Supplementary Fig. 12h, negligible capacitance change was detected down to 1 mm bending radius. Besides, the electrolyte ($PVA\text{-}H_3PO_4$) layer does not show any noticeable capacitance degradation over a year (ΔC = 0.26 mF, Supplementary Fig. 12i).

Supplementary Note 9. Smart textile water resistance. Mobile or wearable electronics directly contacted to human skin are influenced by moisture, water, or sweat during indoor/outdoor activities such as workouts, walking, and running. As we pre-defined that our textile system is not for wearable applications but mainly aiming for indoor smart home applications, washing is not critical. We suggest that our textile system can be dry-cleaned, or air-cleaned (Samsung Air Dresser or LG styler) instead of water-washing.

To further explore practical water resistance of F-devices, we investigated water immersion test under IPX7 condition (1 m deep water, 30 min) based on IEC60529 that reveals the degree of protection against water. After leaving F-LED, F-RF antenna, F-photodetector, F-touch sensor, F-temperature sensor, F-transistor, and F-energy storage devices for 30 min, all F-devices shows unnoticeable performance change including conductivity, current, photocurrent, transfer characteristics, impedance, and total capacitance that satisfy IPX7 requirements (Supplementary Video 9, Supplementary Fig. 25).

Supplementary References

1 Zhang, B. *et al.* Stretchable Conductive Fibers Based on a Cracking Control Strategy for Wearable Electronics. *Advanced Functional Materials* **28**, 1801683, (2018).

2 Lee, S. *et al.* Ag Nanowire Reinforced Highly Stretchable Conductive Fibers for Wearable Electronics. *Advanced Functional Materials* **25**, 3114-3121, (2015).

3 Da Costa Mattos, H. S. *et al.* Analysis of the cyclic tensile behaviour of an elasto-viscoplastic polyamide. *Polymer Testing* **58**, 40-47, (2017).

4 Tran, V.-T., Wei, Y., Yang, H., Zhan, Z. & Du, H. All-inkjet-printed flexible ZnO micro photodetector for a wearable UV monitoring device. *Nanotechnology* **28**, 095204, (2017).

5 Cook, B. *et al.* Inkjet-Printed Imbedded Graphene Nanoplatelet/Zinc Oxide Bulk Heterojunctions Nanocomposite Films for Ultraviolet Photodetection. *ACS omega* **4**, 22497-22503, (2019).

6 Dong, Y. *et al.* An all-inkjet-printed flexible UV photodetector. *Nanoscale* **9**, 8580-8585, (2017).

7 Gong, M. *et al.* All-printable ZnO quantum dots/graphene van der Waals heterostructures for ultrasensitive detection of ultraviolet light. *ACS nano* **11**, 4114-4123, (2017).

8 Guo, Q. *et al.* Sponge-templated production of ultra-thin ZnO nanosheets for printed ultraviolet photodetectors. *Applied Physics Letters* **115**, 122106, (2019).

9 Lin, D., Wu, H., Zhang, W., Li, H. & Pan, W. Enhanced UV photoresponse from heterostructured Ag–ZnO nanowires. *Applied Physics Letters* **94**, 172103, (2009).

10 Liu, B. *et al.* ZnO-nanoparticle-assembled cloth for flexible photodetectors and recyclable photocatalysts. *Journal of Materials Chemistry* **22**, 9379-9384, (2012).

11 Padmanabhan, M., Meyen, R. & Houghton, K. Facile fabrication of ZnO—graphite composite thin films for ultraviolet photodetection. *Materials Research Express* **5**, 095606, (2018).

12 Shao, D., Yu, M., Sun, H., Hu, T. & Sawyer, S. High responsivity, fast ultraviolet photodetector fabricated from ZnO nanoparticle–graphene core–shell structures. *Nanoscale* **5**, 3664-3667, (2013).

13 Tang, R. *et al.* Size-Controlled Graphene Nanodot Arrays/ZnO Hybrids for High-Performance UV Photodetectors. *Advanced Science* **5**, 1700334, (2018).

14 Tran, V.-T., Wei, Y., Zhan, Z. & Du, H. in *Oxide-based Materials and Devices IX.* 105331I (International Society for Optics and Photonics).

15 Wang, H.-C. *et al.* ZnO UV Photodetectors Modified by Ag Nanoparticles Using All-Inkjet-Printing. *Nanoscale Research Letters* **15**, 1-8, (2020).

16 Liang, S. *et al.* ZnO Schottky ultraviolet photodetectors. *Journal of Crystal Growth* **225**, 110-113, (2001).

17 Nie, B. *et al.* Monolayer Graphene Film on ZnO Nanorod Array for High-Performance Schottky Junction Ultraviolet Photodetectors. *Small* **9**, 2872-2879, (2013).

18 Kang, M. *et al.* Graphene-Based Three-Dimensional Capacitive Touch Sensor for Wearable Electronics. *ACS Nano* **11**, 7950-7957, (2017).

19 Takamatsu, S., Yamashita, T., Imai, T. & Itoh, T. Lightweight flexible keyboard with a conductive polymer-based touch sensor fabric. *Sensors and Actuators A: Physical* **220**, 153-158, (2014).

20 Lee, S. *et al.* Nanomesh pressure sensor for monitoring finger manipulation without sensory interference. *Science* **370**, 966-970, (2020).

21 Steinhart, J. S. & Hart, S. R. Calibration curves for thermistors. *Deep Sea Research and Oceanographic Abstracts* **15**, 497-503, (1968).
22 Bayindir, M., Abouraddy, A. F., Arnold, J., Joannopoulos, J. D. & Fink, Y. Thermal-Sensing Fiber Devices by Multimaterial Codrawing. *Advanced Materials* **18**, 845-849, (2006).
23 Scanlon, D. O., Morgan, B. J., Watson, G. W. & Walsh, A. Acceptor Levels inp-TypeCu2O: Rationalizing Theory and Experiment. *Physical Review Letters* **103**, (2009).
24 Barreca, D. *et al.* Chemical vapor deposition of copper oxide films and entangled quasi-1D nanoarchitectures as innovative gas sensors. *Sensors and Actuators B: Chemical* **141**, 270-275, (2009).
25 Ishizuka, S., Kato, S., Maruyama, T. & Akimoto, K. Nitrogen Doping into Cu2O Thin Films Deposited by Reactive Radio-Frequency Magnetron Sputtering. *Japanese Journal of Applied Physics* **40**, 2765-2768, (2001).
26 Shishiyanu, S. T., Shishiyanu, T. S. & Lupan, O. I. Novel NO2 gas sensor based on cuprous oxide thin films. *Sensors and Actuators B: Chemical* **113**, 468-476, (2006).
27 Jiang, C. *et al.* Printed subthreshold organic transistors operating at high gain and ultralow power. *Science* **363**, 719-723, (2019).
28 Han, K.-L. *et al.* Organic/Inorganic Hybrid Buffer in InGaZnO Transistors under Repetitive Bending Stress for High Electrical and Mechanical Stability. *ACS Applied Materials & Interfaces* **12**, 3784-3791, (2019).
29 Hsu, H.-H., Chang, C.-Y. & Cheng, C.-H. A Flexible IGZO Thin-Film Transistor With Stacked TiO_2-Based Dielectrics Fabricated at Room Temperature. *IEEE Electron Device Letters* **34**, 768-770, (2013).
30 Jang, H.-W., Kim, K.-H., Oh, S. & Yoon, S.-M. in *2019 26th International Workshop on Active-Matrix Flatpanel Displays and Devices (AM-FPD).* 1-4 (IEEE).
31 Jeong, H.-J., Kim, B.-S., Han, K.-L., Oh, S. & Park, J.-S. Quantitative analysis of interface trap recovery caused by repetitive bending stress in flexible oxide thin-film transistors. *Japanese Journal of Applied Physics* **58**, 050906, (2019).
32 Kim, Y. C. *et al.* Bending stability of flexible amorphous IGZO thin film transistors with transparent IZO/Ag/IZO oxide–metal–oxide electrodes. *Journal of Alloys and Compounds* **688**, 1108-1114, (2016).
33 Lai, H.-C., Pei, Z., Jian, J.-R. & Tzeng, B.-J. Alumina nanoparticle/polymer nanocomposite dielectric for flexible amorphous indium-gallium-zinc oxide thin film transistors on plastic substrate with superior stability. *Applied Physics Letters* **105**, 033510, (2014).
34 Lee, G.-G. *et al.* The flexible non-volatile memory devices using oxide semiconductors and ferroelectric polymer poly (vinylidene fluoride-trifluoroethylene). *Applied Physics Letters* **99**, 012901, (2011).
35 Lee, G. J. *et al.* High performance, transparent a-IGZO TFTs on a flexible thin glass substrate. *Semiconductor Science and Technology* **29**, 035003, (2014).
36 Ok, K.-C. *et al.* The effects of buffer layers on the performance and stability of flexible InGaZnO thin film transistors on polyimide substrates. *Applied Physics Letters* **104**, 063508, (2014).
37 Park, J.-S. *et al.* Flexible full color organic light-emitting diode display on polyimide plastic substrate driven by amorphous indium gallium zinc oxide thin-film transistors. *Applied Physics Letters* **95**, 013503, (2009).
38 Park, M.-J. *et al.* Improvements in the bending performance and bias stability of flexible InGaZnO thin film transistors and optimum barrier structures for plastic poly

(ethylene naphthalate) substrates. *Journal of Materials Chemistry C* **3**, 4779-4786, (2015).
39 Seo, Y. *et al.* Effect of Simultaneous Mechanical and Electrical Stress on the Electrical Performance of Flexible In-Ga-Zn-O Thin-Film Transistors. *Materials* **12**, 3248, (2019).
40 Spina, F., Costa, J. C. & Münzenrieder, N. in *2019 IEEE International Conference on Flexible and Printable Sensors and Systems (FLEPS).* 1-3 (IEEE).
41 Tripathi, A. K., Myny, K., Hou, B., Wezenberg, K. & Gelinck, G. H. Electrical characterization of flexible InGaZnO transistors and 8-b transponder chip down to a bending radius of 2 mm. *IEEE Transactions on Electron Devices* **62**, 4063-4068, (2015).
42 Xu, H. *et al.* A flexible AMOLED display on the PEN substrate driven by oxide thin-film transistors using anodized aluminium oxide as dielectric. *Journal of Materials Chemistry C* **2**, 1255-1259, (2014).
43 Xu, H. *et al.* Fabrication of flexible amorphous indium-gallium-zinc-oxide thin-film transistors by a chemical vapor deposition-free process on polyethylene napthalate. *ECS Journal of Solid State Science and Technology* **3**, Q3035, (2014).
44 Yoon, J. *et al.* Deep-ultraviolet sensing characteristics of transparent and flexible IGZO thin film transistors. *Journal of Alloys and Compounds* **817**, 152788, (2020).
45 Yu, B.-S. *et al.* Wearable 1 V operating thin-film transistors with solution-processed metal-oxide semiconductor and dielectric films fabricated by deep ultra-violet photo annealing at low temperature. *Scientific reports* **9**, 1-13, (2019).
46 Kou, L. *et al.* Coaxial wet-spun yarn supercapacitors for high-energy density and safe wearable electronics. *Nature communications* **5**, 1-10, (2014).
47 Le, V. T. *et al.* Coaxial fiber supercapacitor using all-carbon material electrodes. *ACS nano* **7**, 5940-5947, (2013).
48 Lee, J. A. *et al.* Ultrafast charge and discharge biscrolled yarn supercapacitors for textiles and microdevices. *Nature communications* **4**, 1-8, (2013).
49 Liu, L., Yu, Y., Yan, C., Li, K. & Zheng, Z. Wearable energy-dense and power-dense supercapacitor yarns enabled by scalable graphene–metallic textile composite electrodes. *Nature communications* **6**, 1-9, (2015).
50 Ma, W. *et al.* Bottom-up fabrication of activated carbon fiber for all-solid-state supercapacitor with excellent electrochemical performance. *ACS applied materials & interfaces* **8**, 14622-14627, (2016).
51 Pech, D. *et al.* Ultrahigh-power micrometre-sized supercapacitors based on onion-like carbon. *Nature nanotechnology* **5**, 651-654, (2010).
52 Ren, J. *et al.* Twisting carbon nanotube fibers for both wire-shaped micro-supercapacitor and micro-battery. *Advanced Materials* **25**, 1155-1159, (2013).
53 Wang, B. *et al.* Fabricating continuous supercapacitor fibers with high performances by integrating all building materials and steps into one process. *Advanced Materials* **27**, 7854-7860, (2015).
54 Xiao, X. *et al.* Fiber-based all-solid-state flexible supercapacitors for self-powered systems. *Acs Nano* **6**, 9200-9206, (2012).
55 Yu, D. *et al.* Controlled Functionalization of Carbonaceous Fibers for Asymmetric Solid-State Micro-Supercapacitors with High Volumetric Energy Density. *Advanced Materials* **26**, 6790-6797, (2014).
56 Yu, D. *et al.* Transforming pristine carbon fiber tows into high performance solid-state fiber supercapacitors. *Advanced Materials* **27**, 4895-4901, (2015).

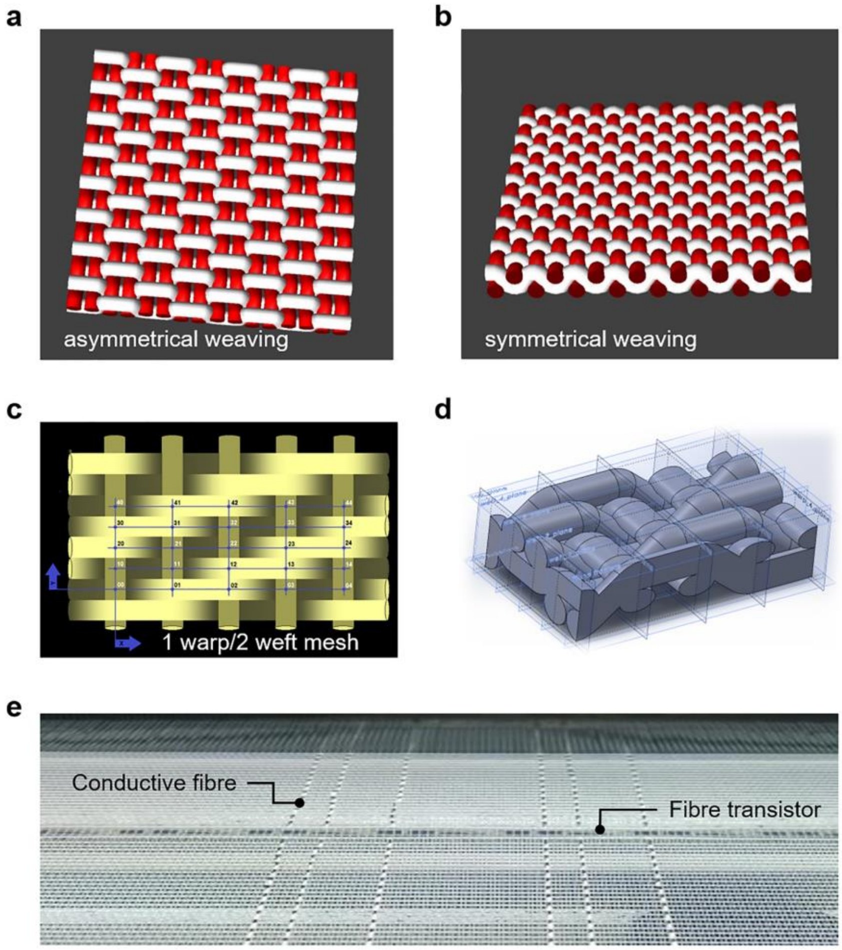

Supplementary Fig. 1.

Weaving pattern design for smart textile system. a, Asymmetrical weaving pattern for electronic devices in smart textile system. **b,** Symmetrical weaving pattern for smart textile frame fabrication. **c,** A schematic illustration of 1 warp/2 weft mesh. **d,** 3D illustration of asymmetrical weaving pattern. **e,** Insertion of F-biosensor module during weaving process and alignment with vertical conductive fiber.

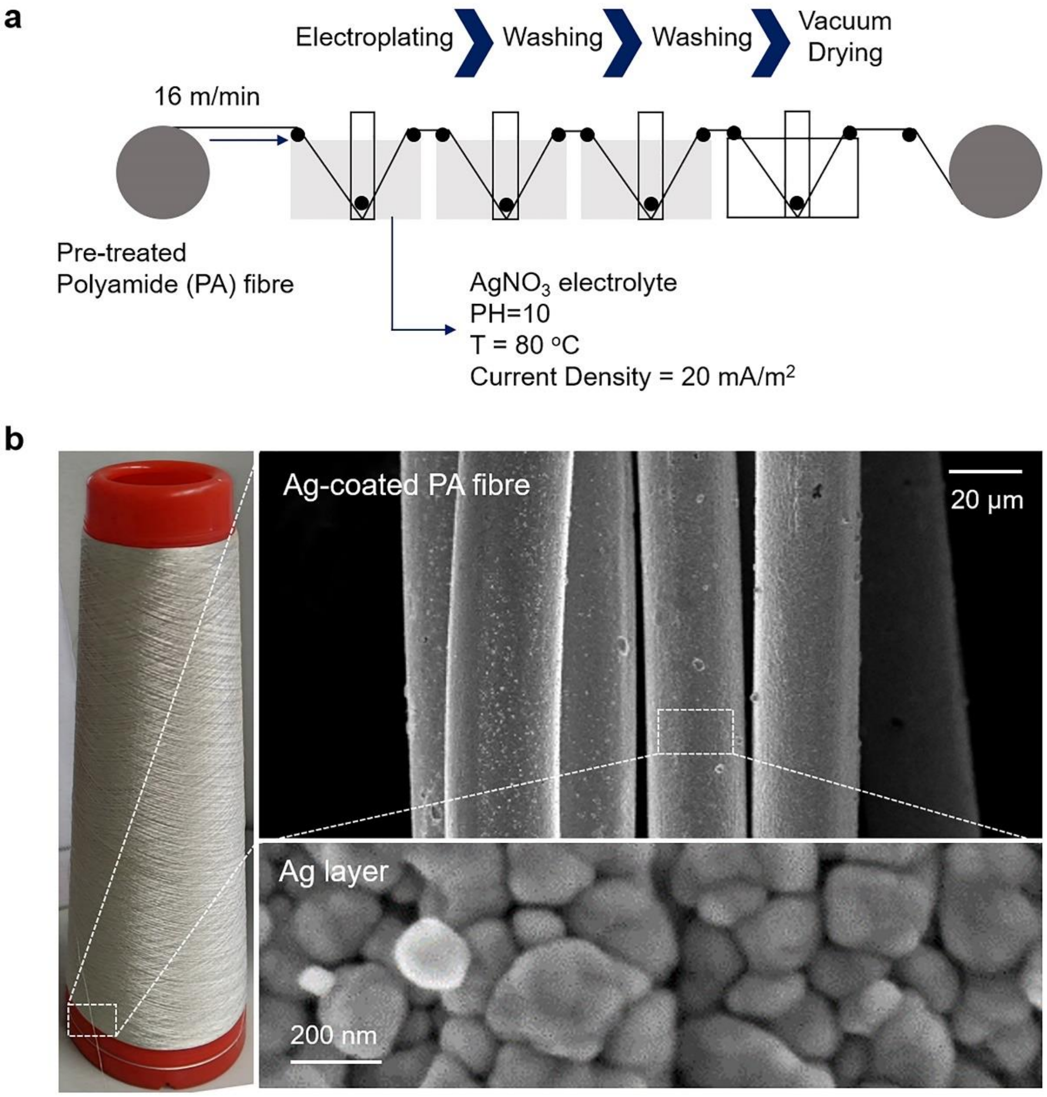

Supplementary Fig. 2.

Fabrication method of conductive fibre. a, The fabrication procedures of conductive fibre; electroplating, washing, and drying. **b,** A photo of 1-km-long conductive fibre and its surface morphology investigated by scanning electron microscope (SEM). Surface of polyamide (PA) fibres is fully coated by silver (Ag) nanoparticles.

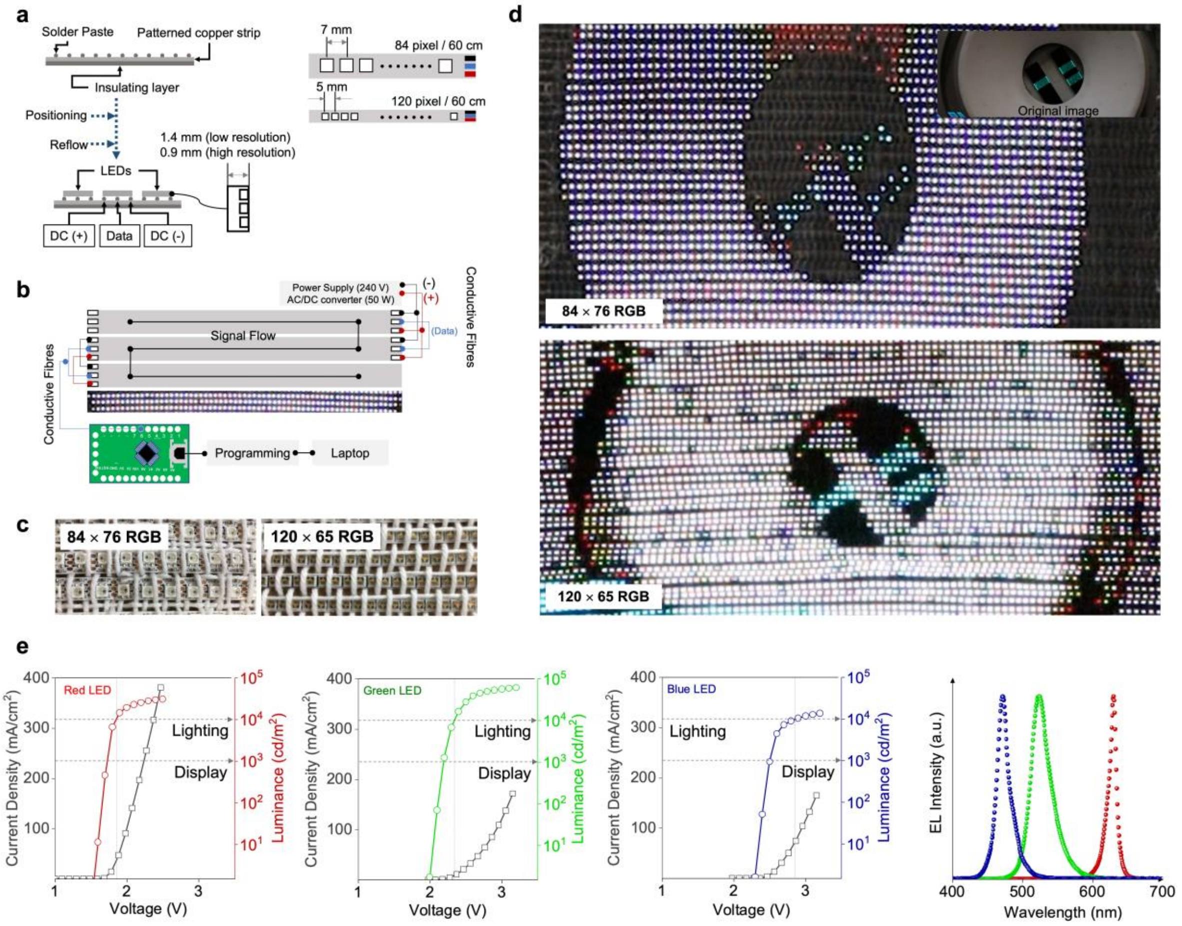

Supplementary Fig. 3.

Fabrication of fibre LED and textile display. a, Fibre LED fabrication method (comparison between high and low resolutions fibres). **b,** The textile display circuit configuration and interconnection method. **c,** Woven F-LED (comparison between low resolution and high resolution). **d,** Comparison of resolution difference depending on number of LED per fibre. **e,** Current density–voltage–luminance (*JVL*) curves and electroluminescence spectra of RGB LED. a. u. on the EL spectra is arbitrary units.

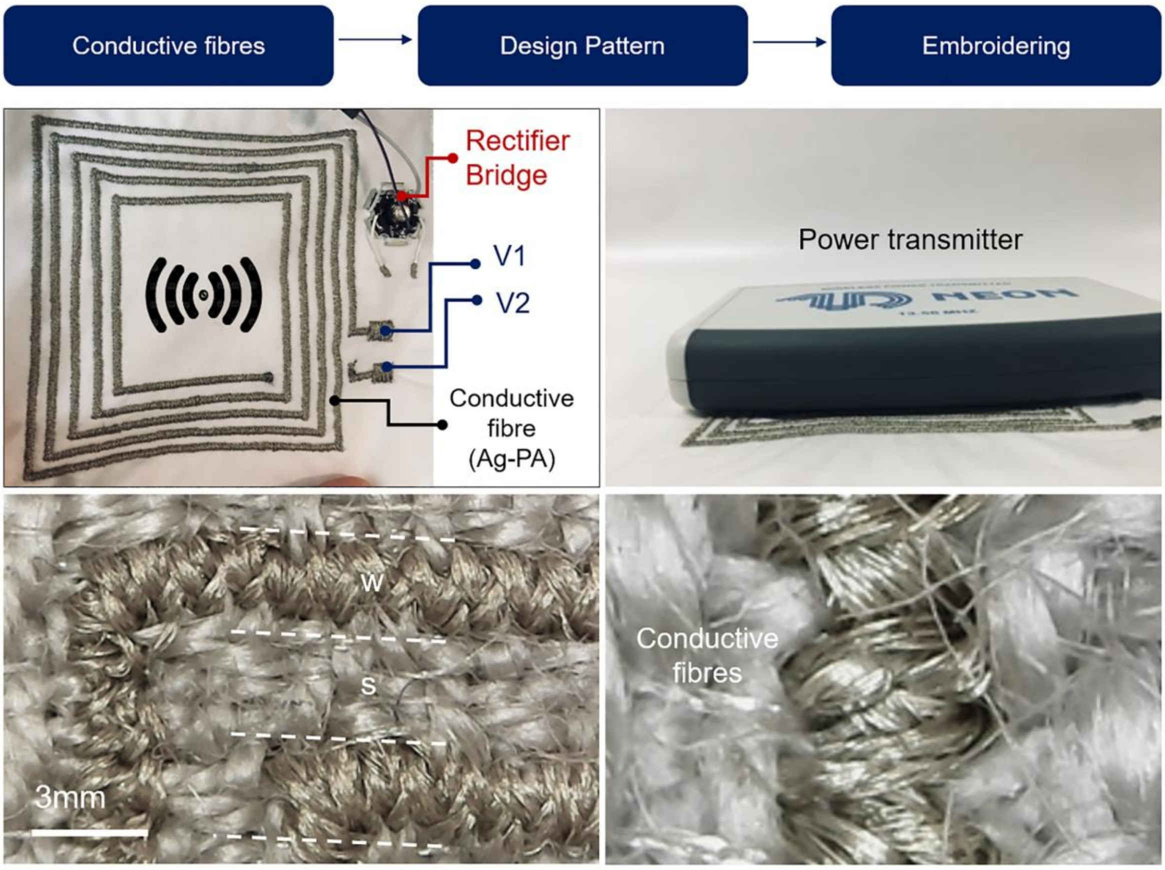

Supplementary Fig. 4.

Photos of F-RF antenna. Conductive fibres (Ag-PA) were used to form an antenna by embroidery method. Designed pattern was delivered to a machine. Rectifier bridge was connected directly to the antenna by conductive fibre (not shown in the photo, the backside of textile). A portable power transmitter was used to test power transferring property. A densely packed conductive antenna is shown in a zoomed photo.

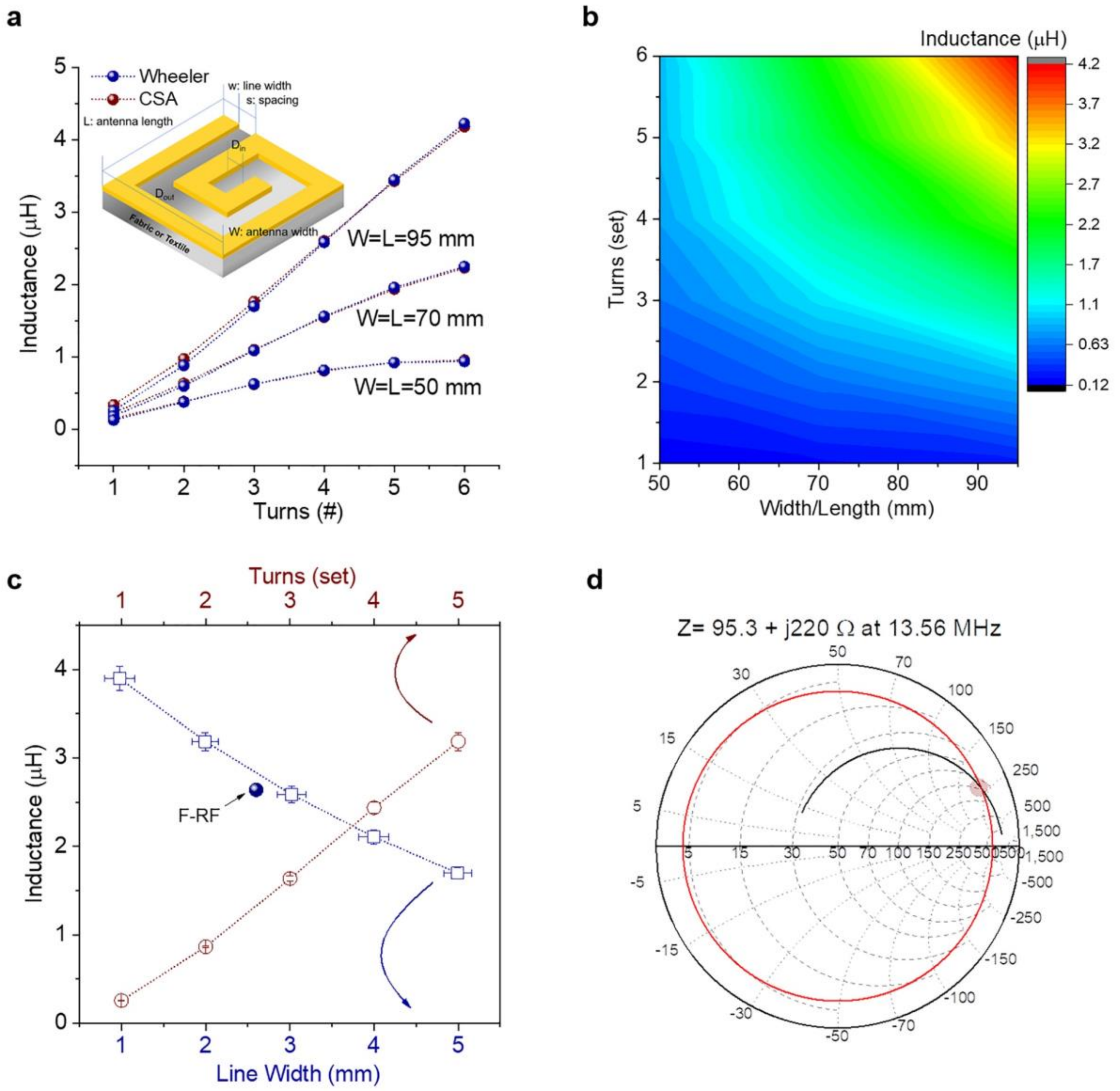

Supplementary Fig. 5.

Calculated inductance of F-RF antenna and measured impedance. a, Calculated inductance of F-RF antenna (variables: dimension and spiral turns). The inset is the schematic description of the dimension of a square spiral antenna. **b,** Inductance density maps calculated by outer length (or width) of antenna and spiral turns, based on the modified Wheeler formula. **c,** Calculated inductance of antenna as a function of line width and turns (blue dot, the measured induction of F-RF). The error bars on the plot represent the standard deviation of calculations. **d,** Measured impedance of F-RF antenna (95.3 + j220 **Ω** at 13.56 MHz).

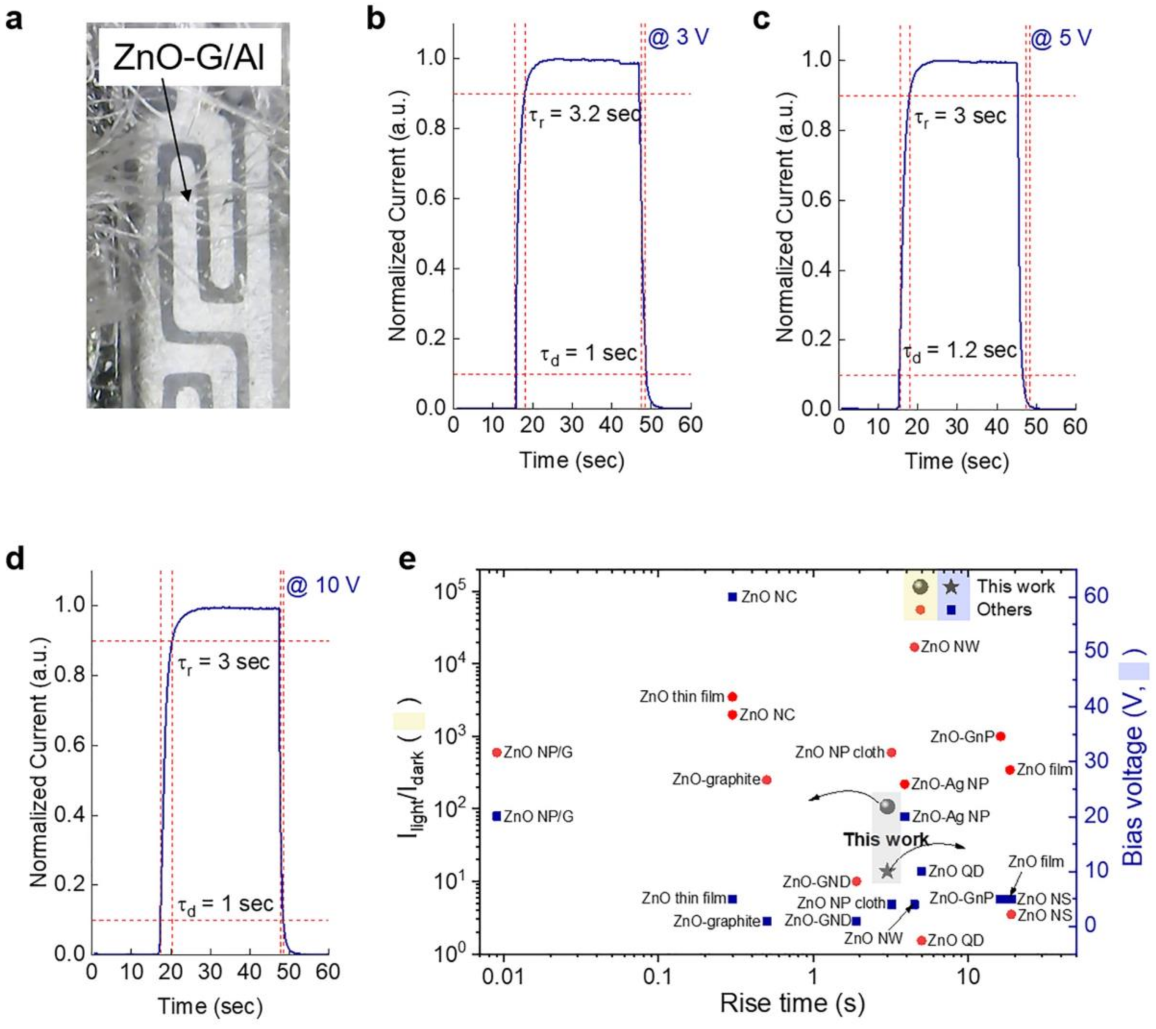

Supplementary Fig. 6.

F-photodetector characteristics. a, A photo of F-photodetector. Extracted current rise and delay at various input voltages; **b,** $\tau_r = 3.2$ and $\tau_d = 1$ sec at 3 V, **c,** $\tau_r = 3$ and $\tau_d = 1.2$ sec at 5V, and **d,** $\tau_r = 3$ and $\tau_d = 1$ sec at 10 V applied to one electrode (the other is grounded). **e,** State-of-the-art flexible ZnO-based photodetector studies. a. u. on the plots is arbitrary units.

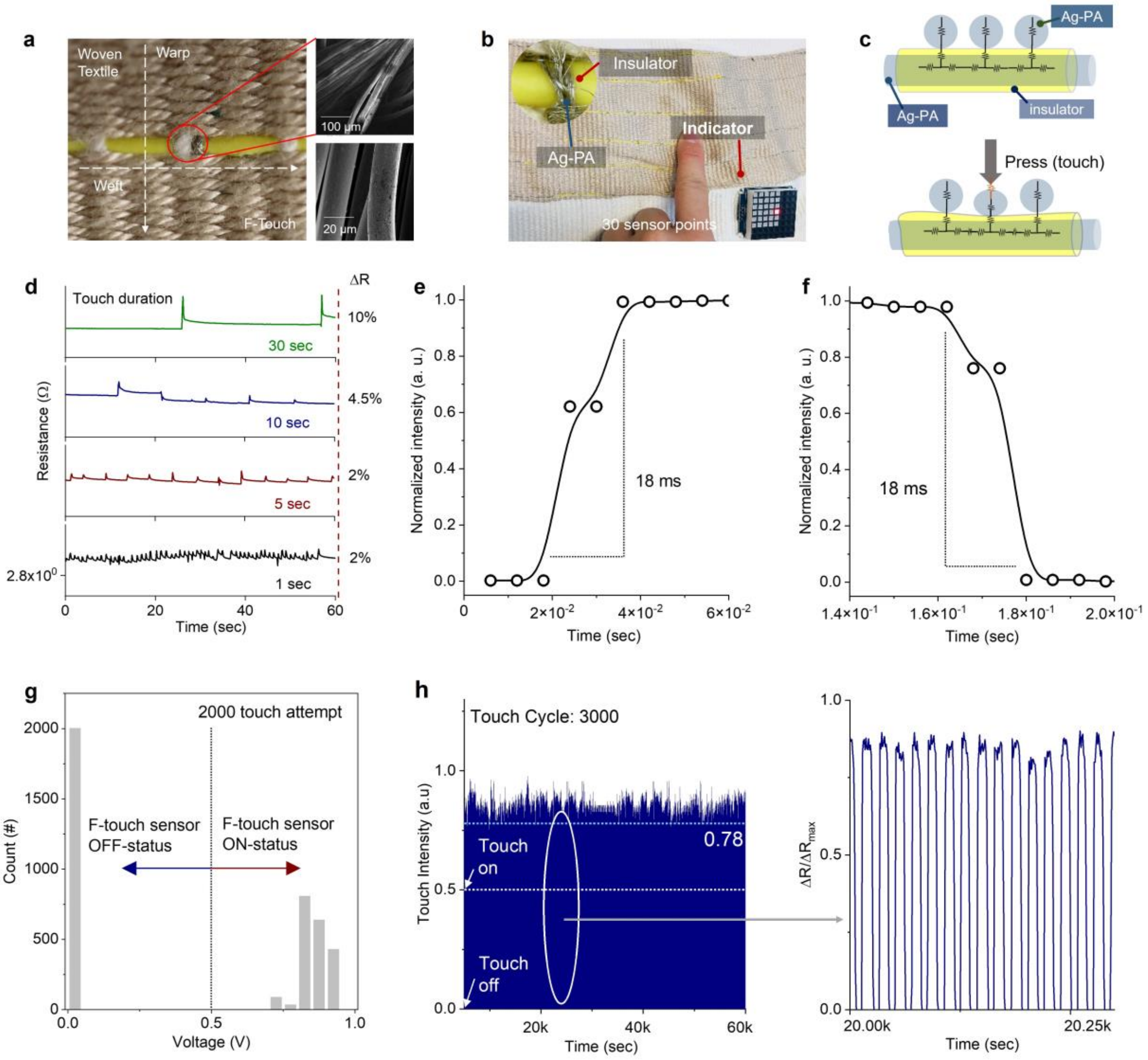

Supplementary Fig. 7.

Fundamentals of F-touch sensor embedded in smart textile system and F-touch sensor response speed and reliability. a, Photo of woven F-touch sensor with cotton/conductive fibres (weft-polymer encapsulation and warp). SEM images show conductive fibres in the textile. **b,** Cross-sectional point of conductive threads. Before connecting to smart textile system, a location-indicative touch sensor was confirmed by connecting 5 × 6 LED matrix. **c,** Working principle of F-touch sensor based on resistance change on each sensor element. **d,** Direct measurement of resistance change without signal processing reveals that a minimal 2% resistance is required to identify the touch as an input stimulus. Relative resistance changes when touch is **e,** Sensed and **f,** Released. Both touch-releasing sensitivities are as fast as 18 ms. **g,** F-touch sensor reliability over 2000 touch attempts. On-voltage was programmed at 0.5 V. Above 0.5 V, touch attempt is believed to be solid input. **h**, Touch attempts up to 3000 cycles to validate the resistance against surface fatigue by human finger. a. u. on the plots is arbitrary units.

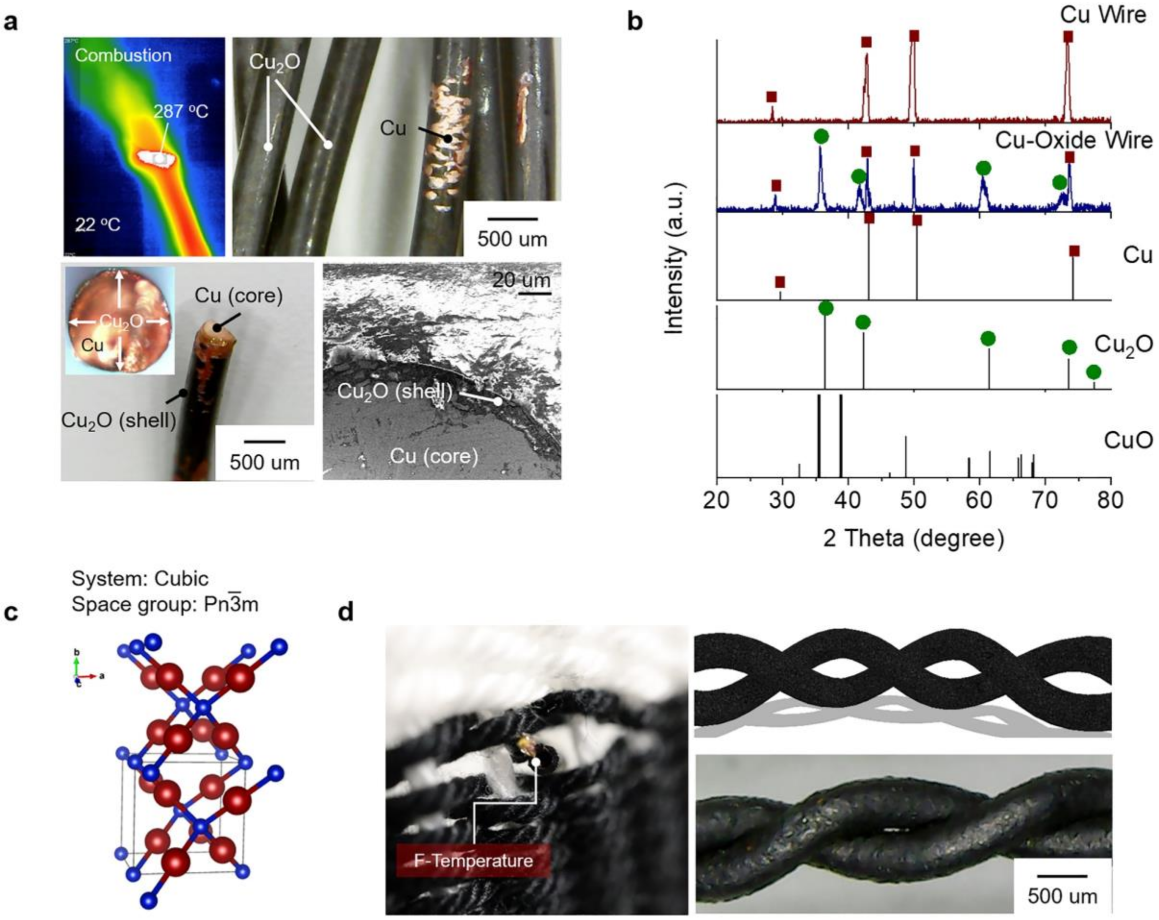

Supplementary Fig. 8.

Fabrication method and woven F-temperature sensor. a, infrared photo during the combustion oxidation of Cu fibre to form a Cu_2O. **b,** XRD pattern of Cu/Cu_2O fibre. a. u. on the plot is arbitrary units. **c,** Crystal structure of Cu_2O. **d,** Twisted Cu/Cu_2O F-temperature sensor and embedded as a replacement of weft (horizontal) thread.

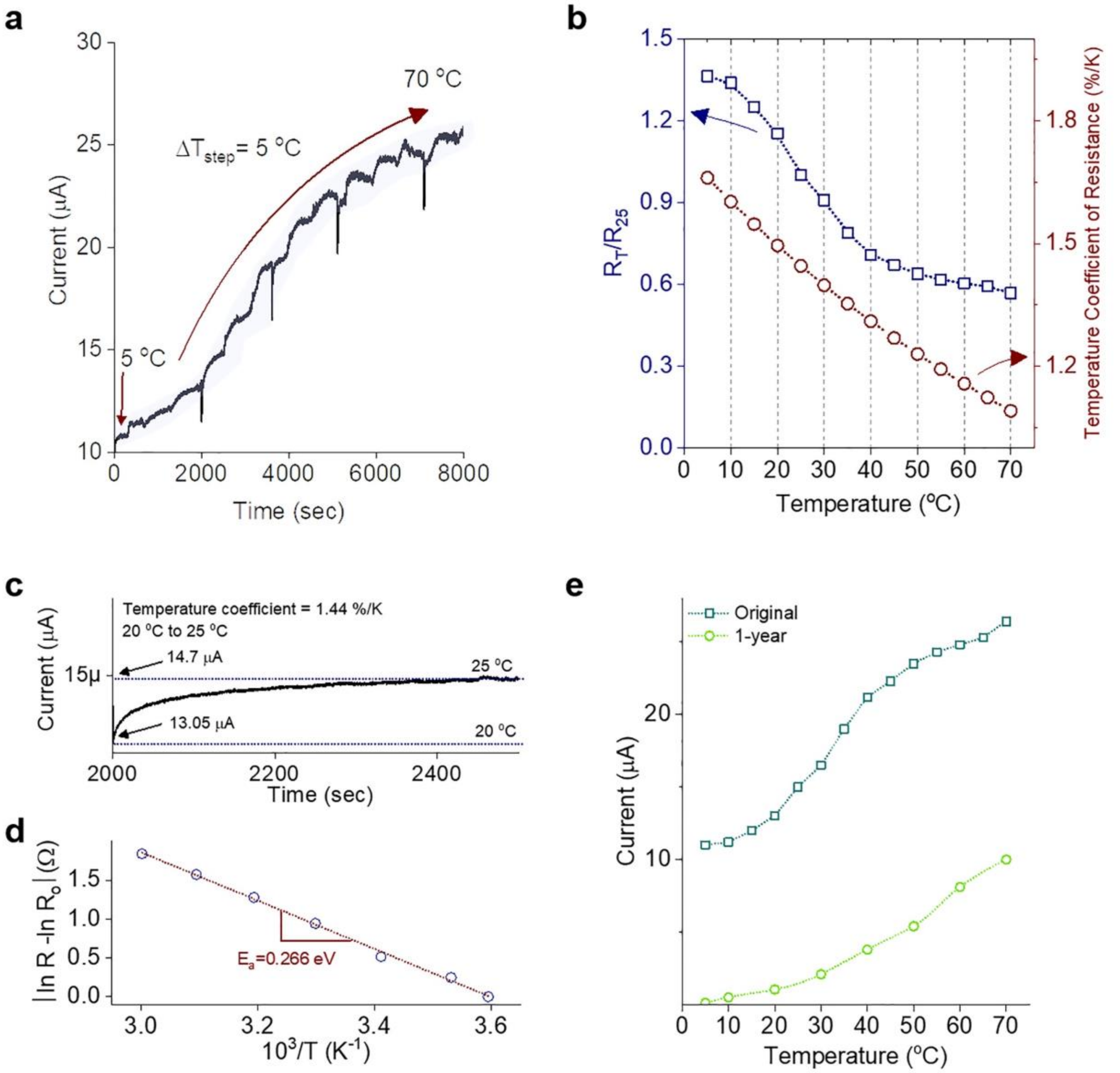

Supplementary Fig. 9.

Characteristics of F-temperature sensor. a, Measured current as a function of temperature (at 5 °C steps, heat source: a hot plate with accurate temperature readout). **b,** Extracted temperature coefficient of resistance and normalised resistance change (R_{25} is reference condition). **c,** Temperature sensitivity estimation at 25 °C (1.44 %/K). **d,** Extracted activation energy (0.266 eV, range from 5 °C to 60 °C). **e,** Stability of F-temperature sensor over a year.

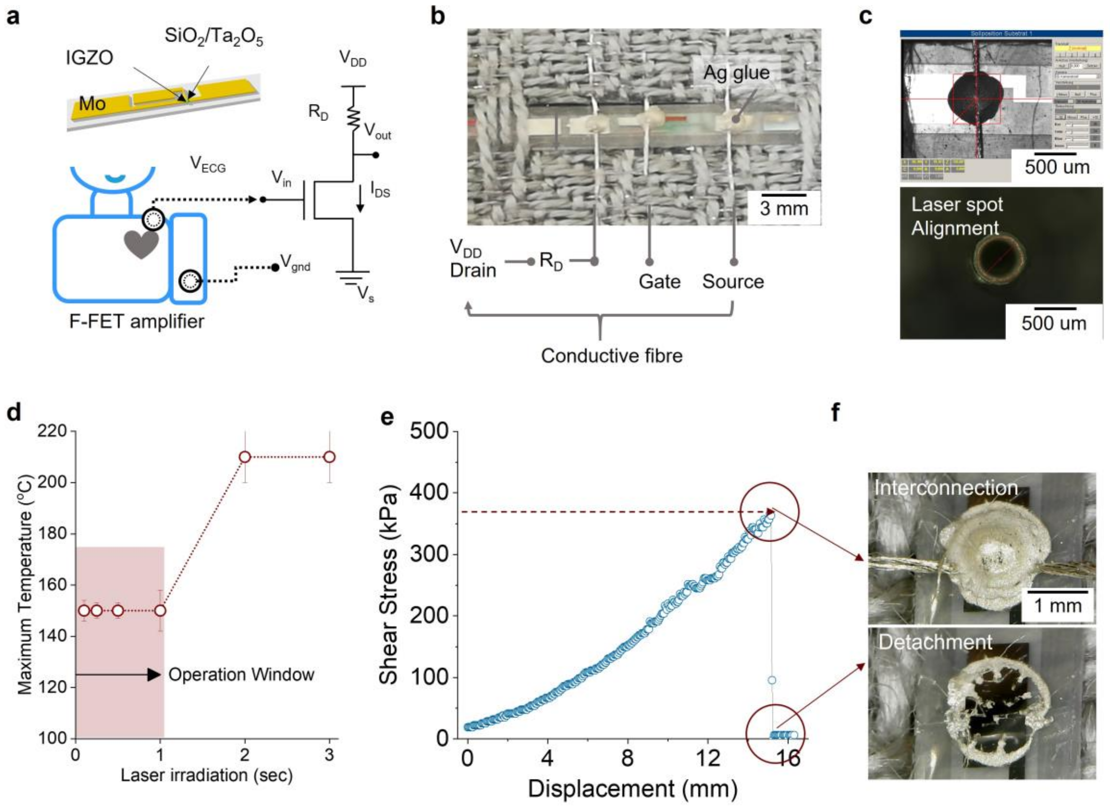

Supplementary Fig. 10.

A schematic illustration of F-biosensor module and its interconnection within cotton textile with conductive threads. a, Schematic illustration of IGZO transistor and amplifier circuit diagram for heartbeat detection. **b,** Interconnection between conductive threads and transistor electrodes (gate, source, and drain). **c,** Laser soldering procedure and photos of real-time monitoring during laser irradiation. **d,** Temperature profile of Ag glue as a function of laser irradiation time. The error bars on the plot represent the standard deviation of measurements. **e**, Strength of laser-soldered Ag glue holding a conductive fibre. **f**, Photos of Ag glue on drain electrode of IGZO transistor before/after shear stress test.

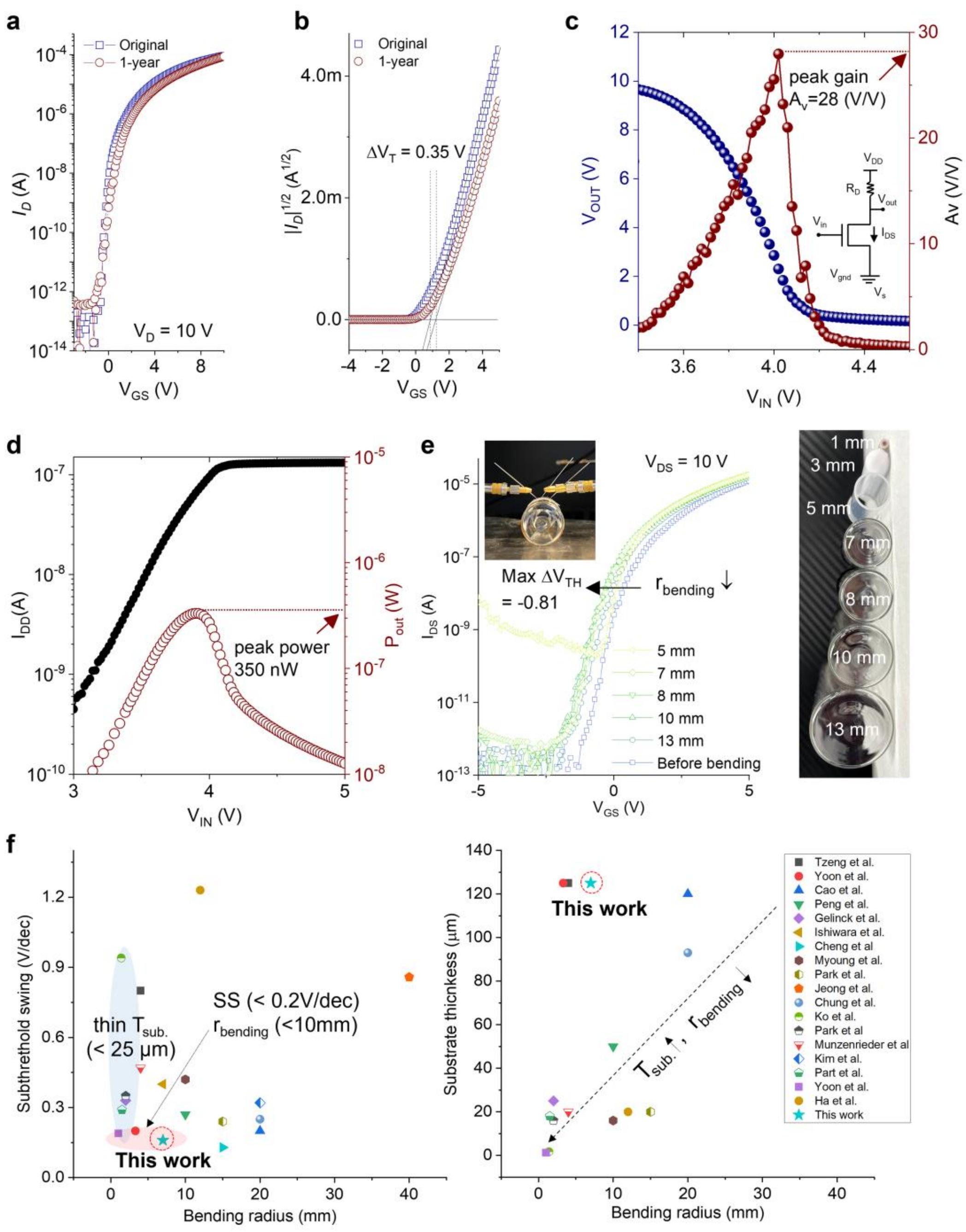

Supplementary Fig. 11.

Characteristics of F-biosensor module and state-of-the-art of flexible IGZO transistor. a, Transfer characteristics and electrical stability of IGZO transistor over a year. **b,** Threshold voltage shift over a year. **c,** Measured voltage gain (A_v = 28). **d,** Current and power consumption of F-biosensor module. **e,** Photo of bending test conditions and measurement setup. Critical assessment of bending conditions for F-transistor as a function of transfer characteristics. **f,** State-of-the-art flexible IGZO transistor studies focusing on high mechanical stability show that our transistor exhibits comparable bending stability even with a thicker substrate.

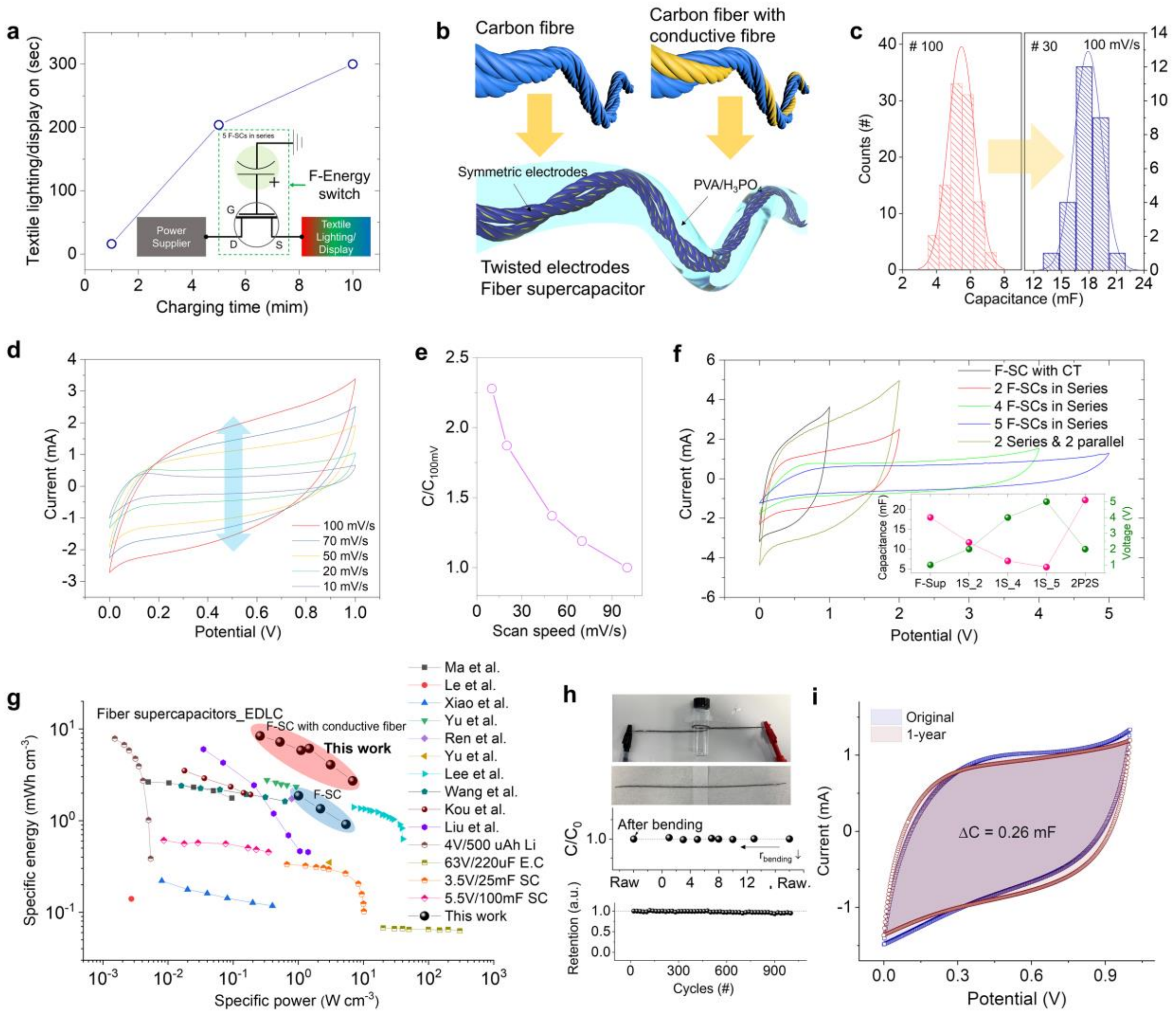

Supplementary Fig. 12.

F-energy storage as textile display power switch and improved capacitance by incorporating conductive fibre. a, F-energy storage as a power switch of textile display. **b,** A schematic illustration of conductive fiber with carbon bundle for F-supercapacitor electrodes. **c,** Histogram of capacitance distribution (3-fold increase with conductive fiber). **d-e,** CV characteristics of fibre supercapacitor on various scanning speeds. **f,** Serial/parallel connection of F-energy device for higher voltage output and higher capacitance. **g,** Ragone plots compared with the selected previous fiber-shaped supercapacitor (electrical double layer capacitor). **h,** Photos of fibre supercapacitor and bent device. The constant capacitance of fibre supercapacitor under bending radius more than 1 mm and electrochemical stability of F-energy storage under 1000 cyclic charge-discharge test. **i,** Conservation of initial capacitance over a year ($\Delta C = 0.26\ mF$).

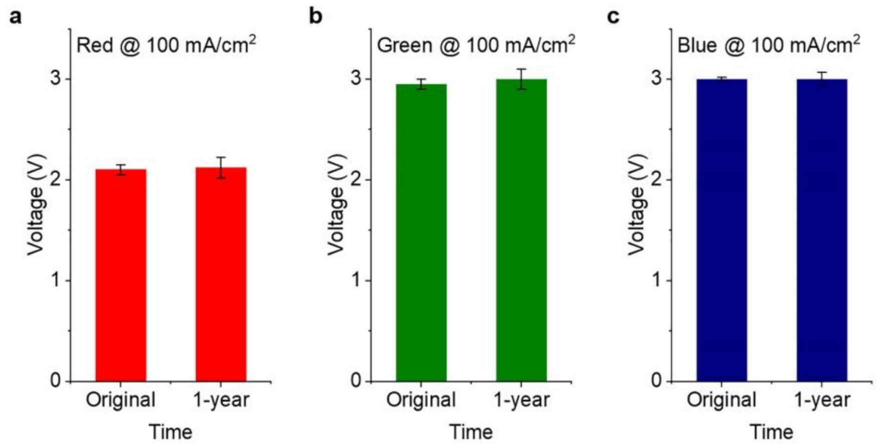

Supplementary Fig. 13.

Electrical (operating voltage at 100 mA/cm^2) and mechanical (bending radius at 20 mm) stability of F-LED over a year. Operating voltage at 100 mA/cm^2 over a year; **a,** red, **b,** green, and **c,** blue. The error bars on all the bar graphs represent the standard deviation of measurements.

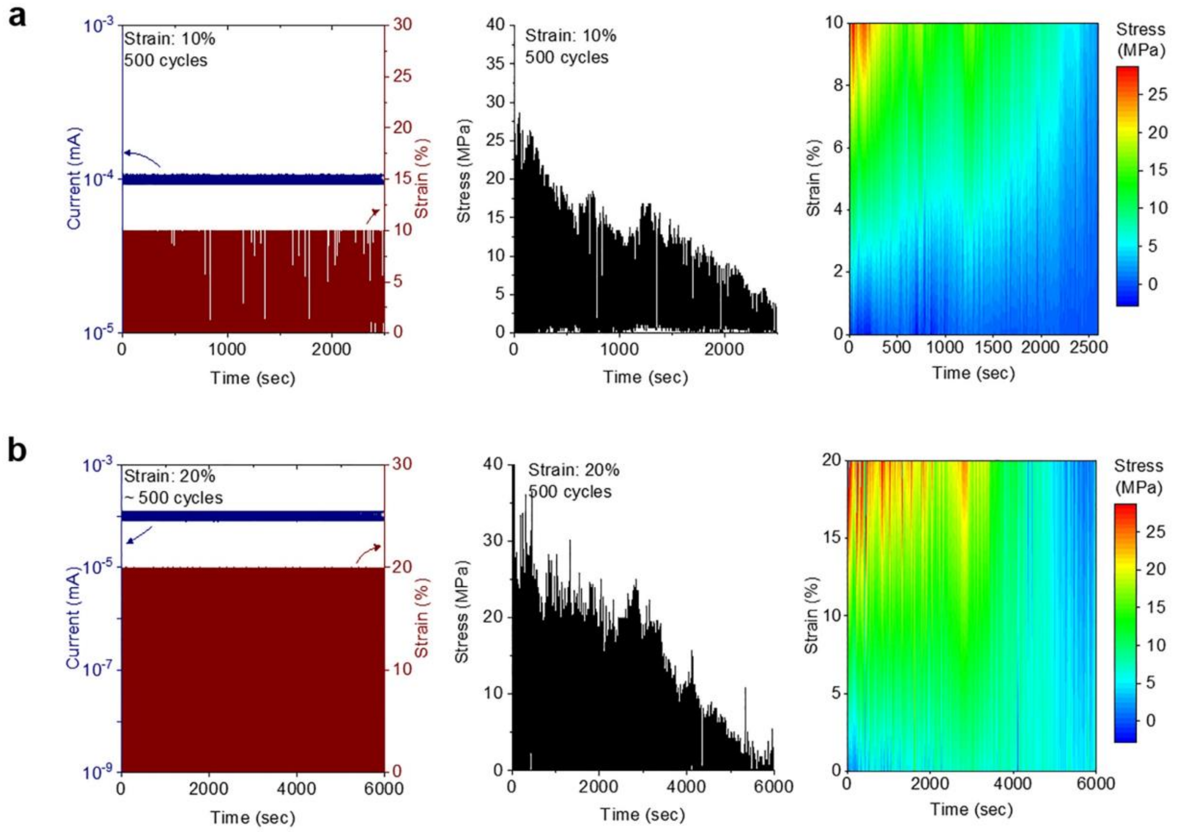

Supplementary Fig. 14.

Mechanical stability of conductive fibre (Ag-PA). a, Cyclic bending test under 10% strain of conductive fibre showing initial plastic deformation. **b,** Cyclic bending test under 20% strain of conductive fibre showing continuous plastic deformation.

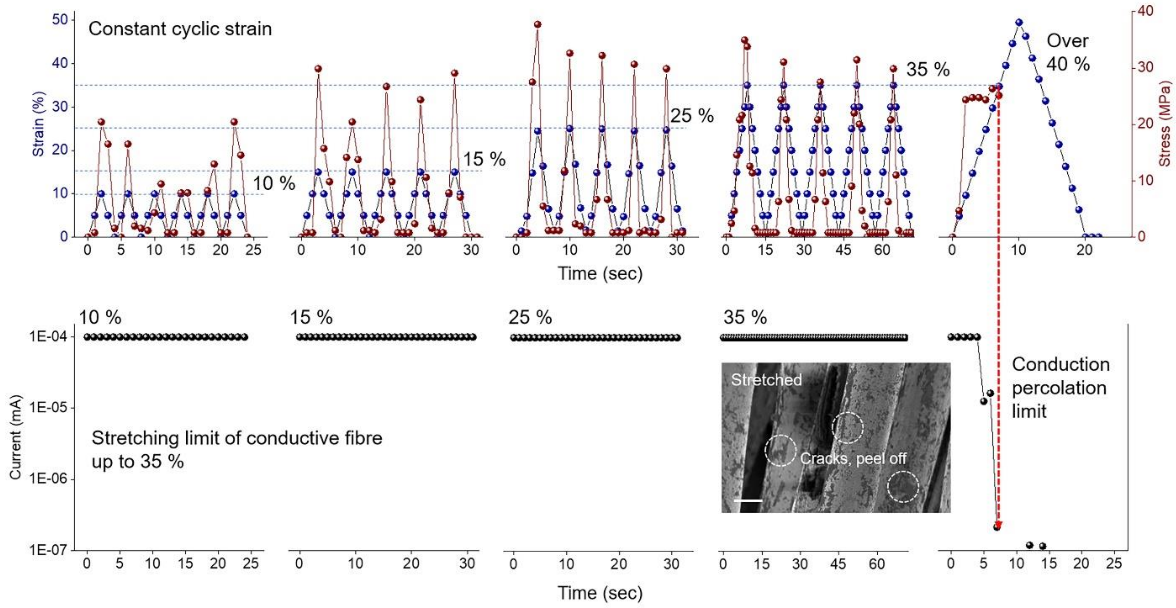

Supplementary Fig. 15.

Mechanical properties of conductive fibres (Ag-PA). Cyclic strain applied to conductive fibres (78 dtex: grams per 10 km of yarn, f18: the number of filaments). Two-electrode current measurements indicate that conductivity of up to 35% strain is stable and above which the Ag layer delaminates. SEM image in the inset shows surface morphology of conductive fibres after stretching (scale bar: 10 µm).

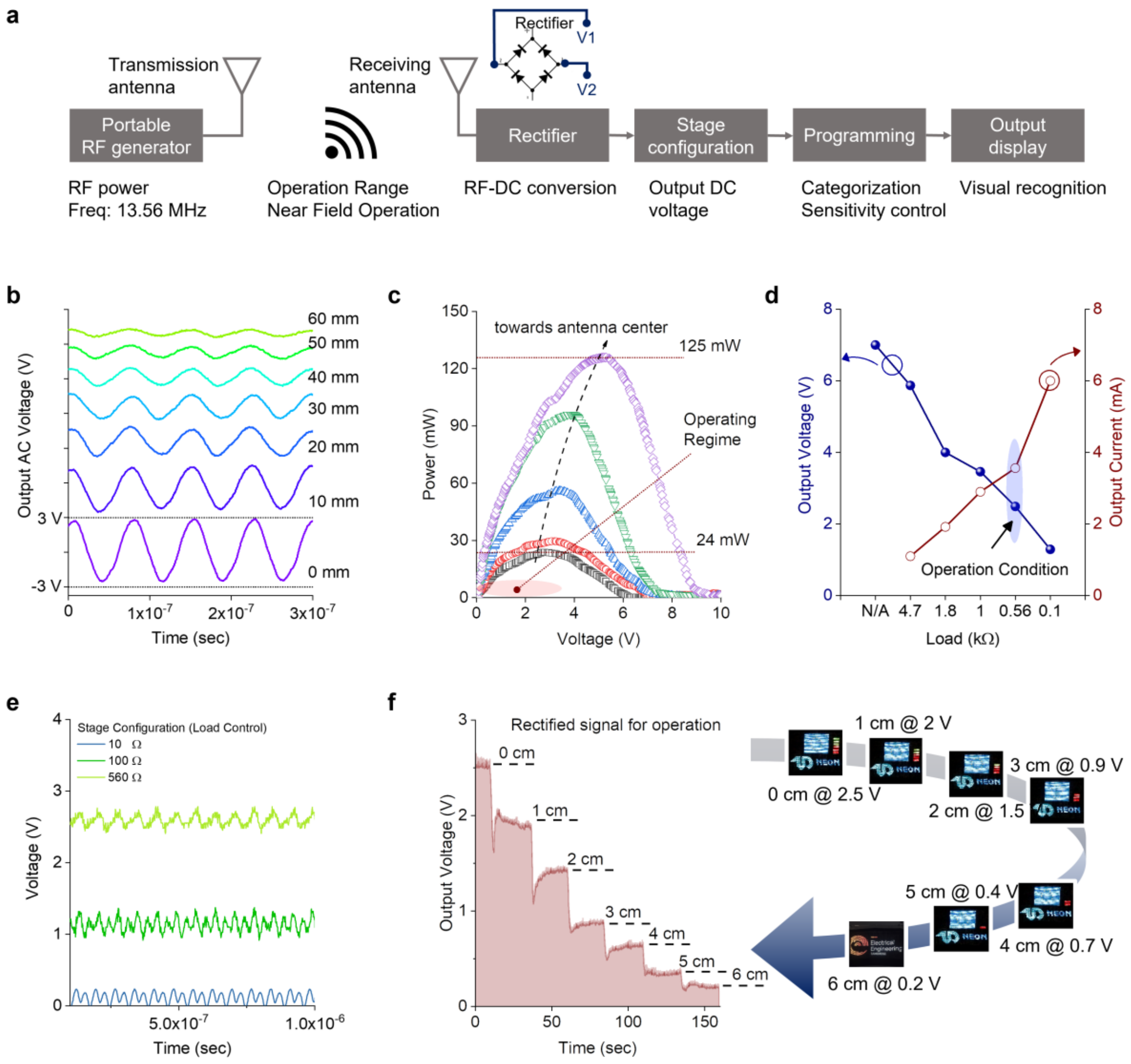

Supplementary Fig. 16.

Characteristics of square spiral F-RF antenna. a, A schematic illustration of F-RF antenna operation scheme. Stage configuration was down by categorising output voltage. We have divided 1 cm of distance sensitivity by programming code. **b,** Without a rectifier, AC voltage is directly measured from the antenna. **c,** Output power as a function of distance from a portable RF generator to the antenna. **d,** Matching a load with the antenna for minimum power consumption and higher signal sensitivity. **e,** Rectified voltage with suppressing load resistor (10, 100, and 560 Ω). **f,** Signal classification of output voltage and representative images displaying on the F-LED depending upon distance.

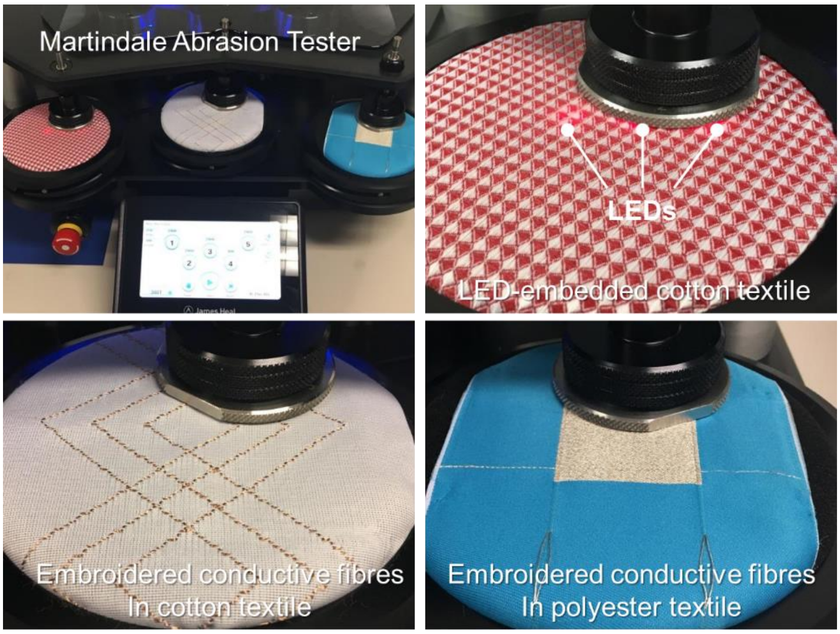

Supplementary Fig. 17.

Abrasion test (Martindale method) for functional textiles. Cyclic abrasion of textile with disc-type surface tester was applied onto RGB LEDs and conductive fibres embroidered both on cotton and polyester textiles at a fixed load of 1 kg. After 1000 cycles, the luminance of LEDs maintains 1000±10 cd/m^2 at a given voltage (1.75 V) as well as the conductivity of Ag-PA fibres shows unnoticeable change ($2.32\times10^6\pm0.5$ S/m) which validates the mechanical stability of F-devices.

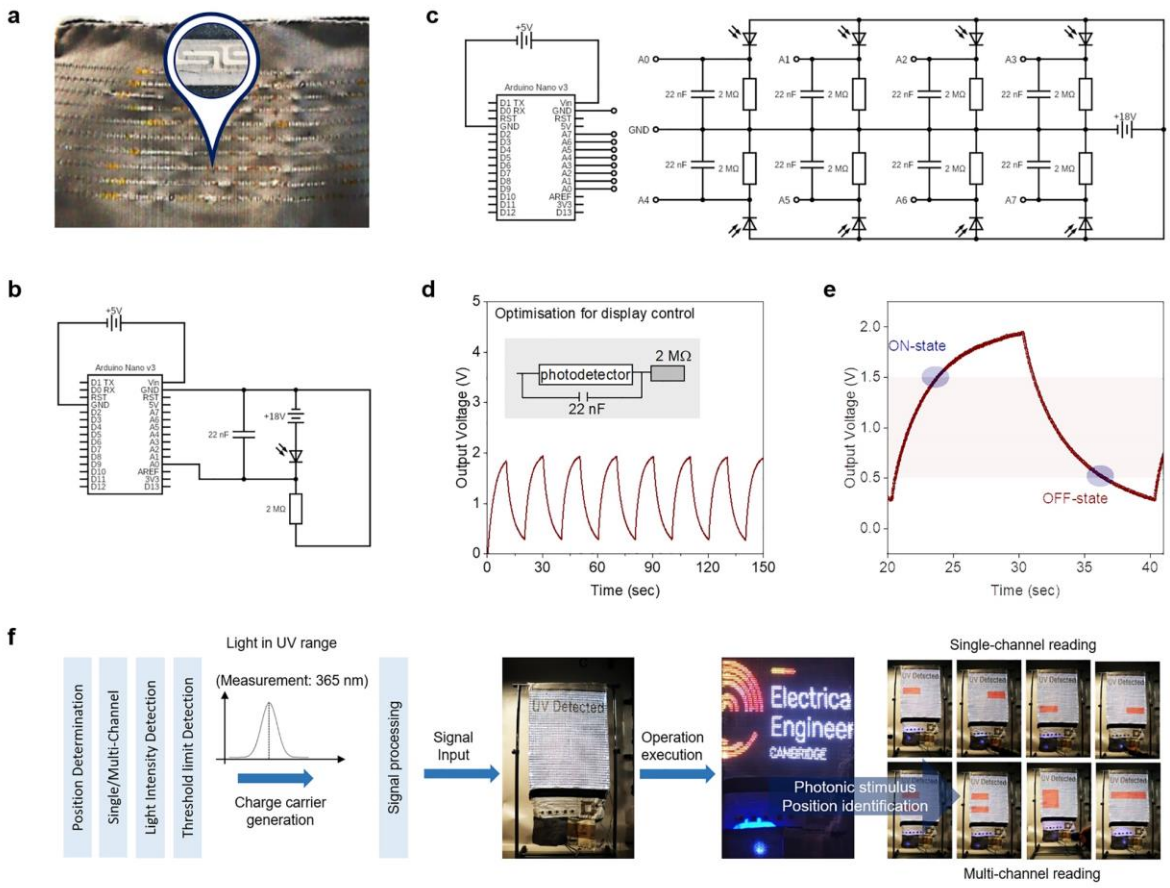

Supplementary Fig. 18.

Operating principle by F-photodetector. a, Photo of woven F-photodetector in cotton fabric. All F-photodetectors are aligned in horizontal direction to minimize mechanical damage during the weaving process. **b,** Single F-Photodetector circuit for operating test. **c,** Eight-channel F-photodetectors installed in the smart textile system in this study. **d,** Output voltage of F-Photodetectors installed in the system. The operation condition (t_{UV} = 10 sec) was optimised for visualising the UV detection function on the textile display. **e,** Classification of on-off status based on the output voltage. ON/OFF states are programmed at output voltages of 1.5 V and 0.5 V, respectively. **f,** Schematic of UV photodetector signal processing sequence. Working principle of F-photodetector and photos of non-activated smart textile (idle status)/activated status (university logo) or position-dependent activated status in the eight-channel array.

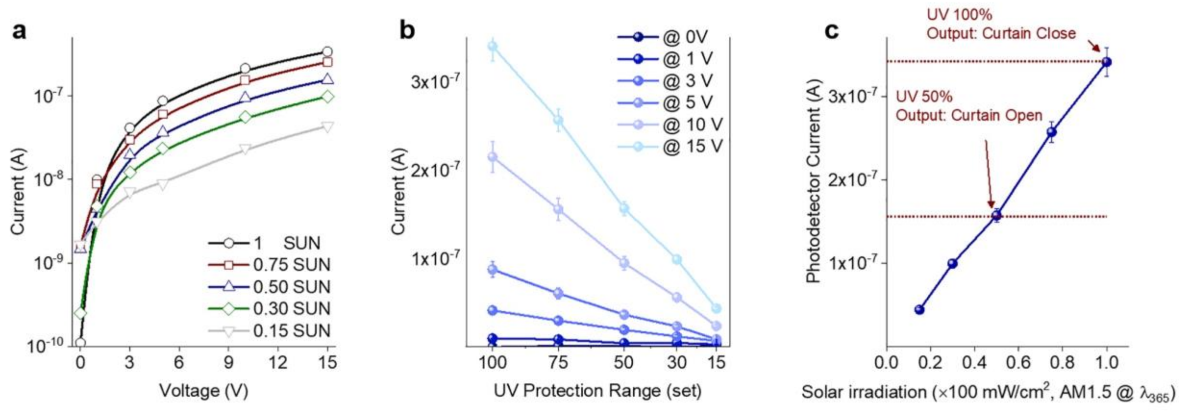

Supplementary Fig. 19.

Implementation of F-photodetector in real-life application (smart curtain). a, I-V characteristics under various solar irradiation conditions (AM 1.5G). **b,** Classification of current range with UV filter located on F-photodetector. **c,** Simulated smart curtain operation by detected UV range (opening smart curtain is set to 50% of UV irradiation measured at 385 nm). The error bars on the plots (**b** and **c**) represent the standard deviation of measurements.

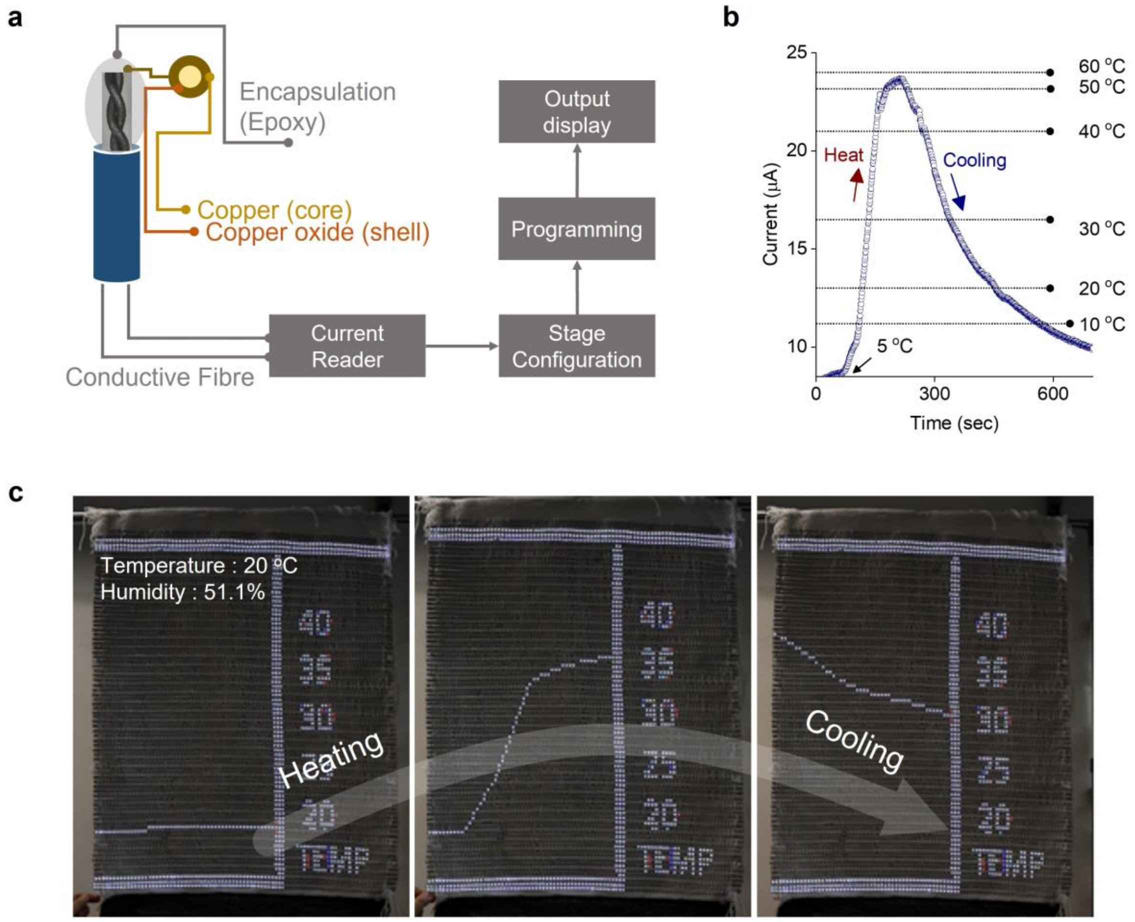

Supplementary Fig. 20.

Fabrication method and weaving/circuitry of F-temperature sensor. a, Schematic illustration of encapsulated F-temperature sensor and its circuitry with current reader connected to F-LED display. **b,** Fast heating of F-temperature sensor on a hot plate. **c,** Real-time monitoring of temperature (grasping F-temperature sensor by fingers) displayed on F-LED.

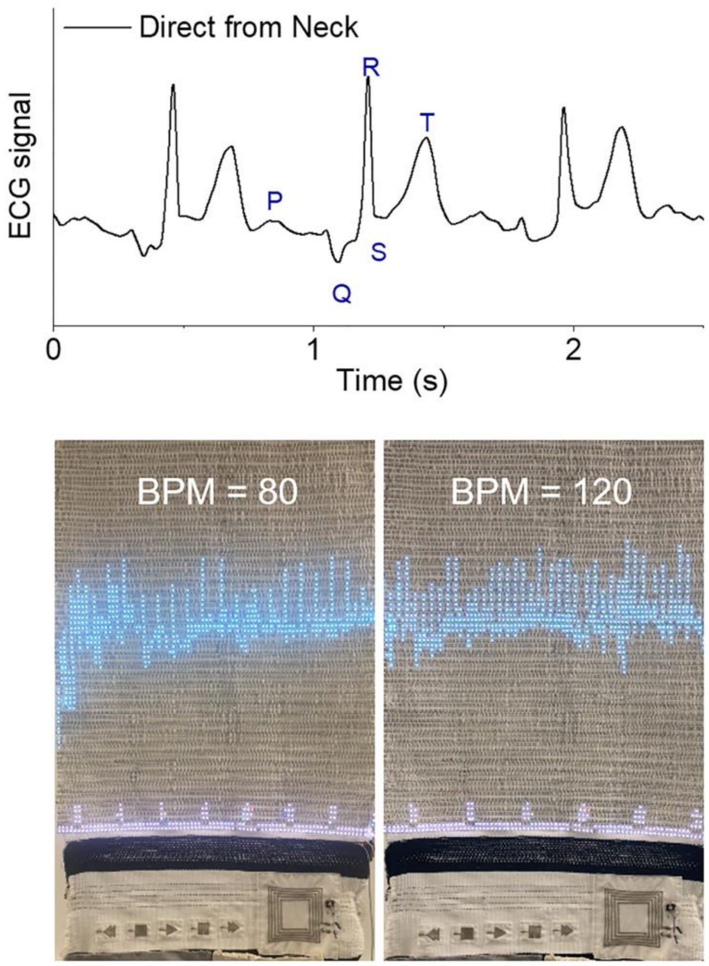

Supplementary Fig. 21.

Displaying amplified ECG signal on the smart textile system. Measured ECG signal from a person and captured photos of the smart textile system during F-biosensor module operation with different heartbeat rate (before/after running).

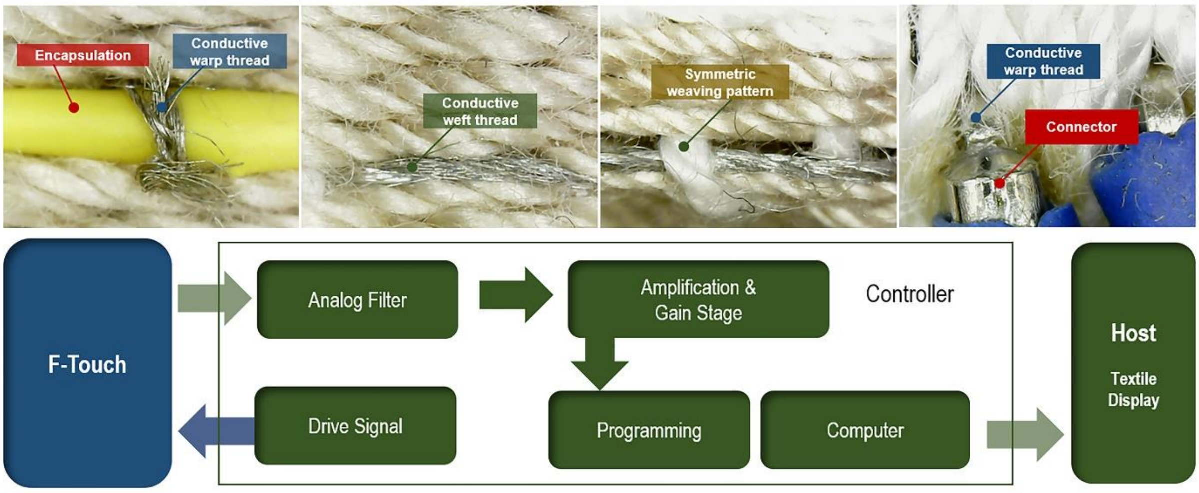

Supplementary Fig. 22.

Interconnection method from F-touch sensor to controller and flow diagram of signal processing. A Conductive fibre has approximately 20 filaments in one thread. Symmetrically woven F-touch sensor textile is connected to a controller by using a pin-connector.

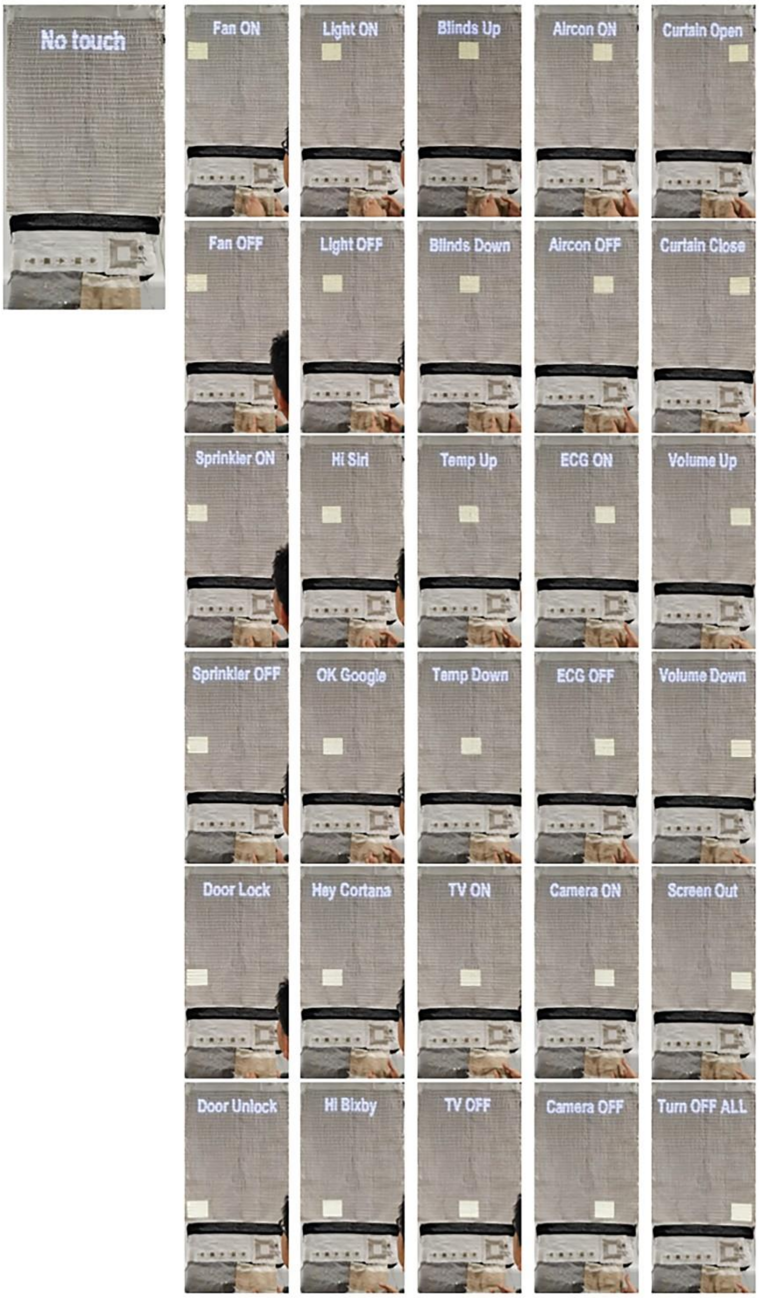

Supplementary Fig. 23.

Smart home gadget communicating with F-touch sensor. This photo summarises all 30 touch elements coded for smart home-appliance operation or voice/video recognition functions.

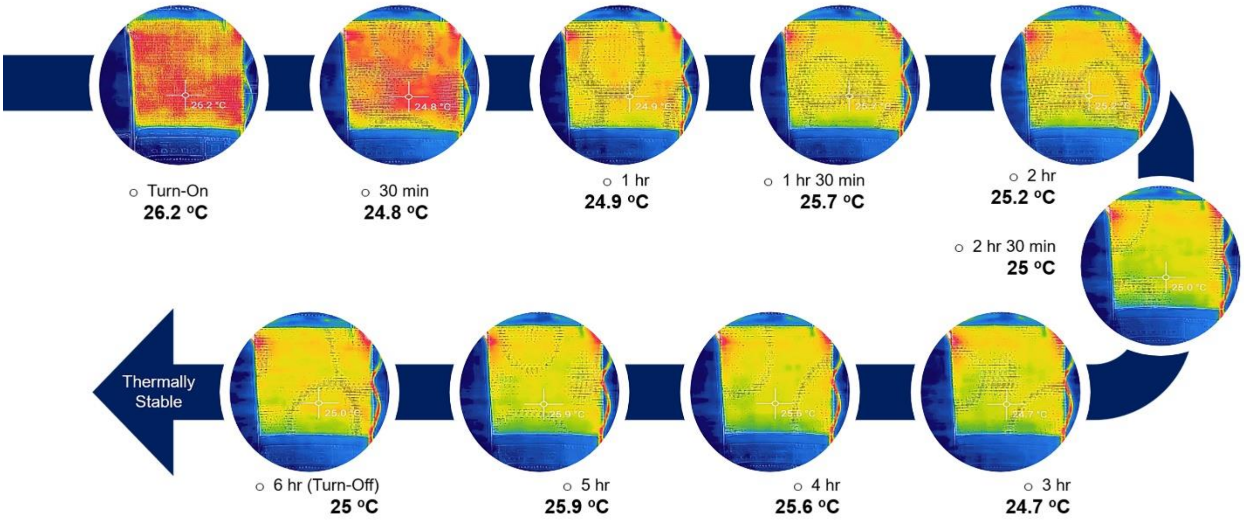

Supplementary Fig. 24.

Thermal stability of textile system. Continuous running of textile display for 6 hours leads to temperature variation less than 2 ^{o}C (from 24.7 to 26.2 ^{o}C), which is highly safe for human interaction (touch or structural/physical transformation). Excluding F-LED module, all other fiber-based devices remain their temperature below 24 ^{o}C as confirmed by the infrared camera (both Seek thermal and FLIR camera).

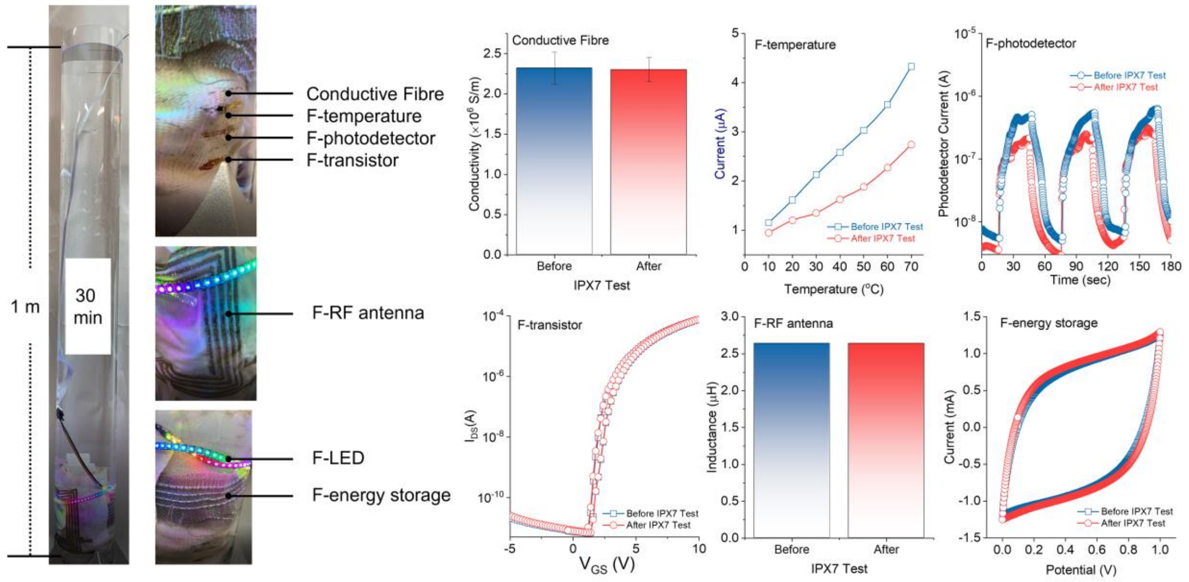

Supplementary Fig. 25.
Standardized water resistance test (IPX7, 1m deep, 30 min) for all F-devices including F-LED, conductive fibre**,** F-temperature sensor, F-photodetector, F-transistor, F-RF antenna, F-energy storage (supercapacitor) that shows unnoticeable performance change after immersion in the water for 30 min. The error bars on the bar graph represent the standard deviation of measurements.

Supplementary Table 1.

Conductive fibre fabrication speed as a function of drive frequency

Drive Frequency (Hz)	Thread Running Speed (m/min)
5	2.1
10	4.2
15	6.02
20	8.27
25	10.37
30	12.31
35	13.02
40	16

Supplementary Table 2.

Deposition parameters of conductive fibre during electroplating

**parameters**	**minimum**	**Maximum**
Speed	1 m/min	16 m/min
Temperature	25 ^{o}C	80 ^{o}C
Volume of electrolyte	100 mL	80 L
Current density	0.5 A/0.01 m^2	2 A/0.01 m^2
Layer thickness	100 nm (±50%)	5 μm (±10%)
fibre count	1 (Φ = 50 μm)	64 (Φ = 20 μm)

Supplementary Table 3.

Summary of parameters for F-RF antenna inductance simulation.

Parameters	Value
Turns	Variable
Total Length (mm)	Variable
Total Width (mm)	Variable
Line width	Variable
Line spacing	2, 2.5 mm
Line height (mm)	500 µm
Substrate Thickness	700 µm
Substrate Permittivity	3.18
K_1 (square)	2.34
K_2 (square)	2.75
c_1 (square)	1.27
c_2 (square)	2.07
c_3 (square)	0.18
c_4 (square)	0.13

Supplementary Table 4.

Material property of Ag conductive glue for laser soldering

Component(s)	**CAS Number**	**Percentage**
Silver	7440-22-4	80-90
Fatty acids, C18-unsatd., dimers, hydrogenated, di-Me esters, hydrogenated, bis(2,5-dihydro-2,5-dioxo1H-pyrrole-1-hexanoate)		1-5
Isobornyl acrylate	5888-33-5	1-5
2,2-dimethyl-1,3-propanediyl bismethacrylate	1985-51-9	1-5
1,3-Butadiene-maleic anhydride copolymer	25655-35-0	1-5
Formaldehyde polymer with 1,1'oxybis[benzene],2-methyl-2- propenoate		0.1-1
(2,4,6-Trioxo-1,3,5-triazine1,3,5(2H,4H,6H)-triyl)tri-2,1-ethanediyl triacrylate	40220-08-4	0.1-1

Supplementary Table 5.

F-touch sensor execution code for smart home applications.

Weft \ Warp	1	2	3	4	5
1	Fan On	Light On	Blinds Up	Aircondition On	Curtain Open
2	Fan Off	Light Off	Blinds Down	Aircondition Off	Curtain Close
3	Sprinkler On	Hi Siri	Temp UP	ECG On	Volume Up
4	Sprinkler Off	OK Google	Temp Down	ECG Off	Volume Down
5	Door Lock	Hey Cortana	TV On	Camera On	Screen Out
6	Door Unlock	Hi Bixby	TV Off	Camera Off	Turn Off All
