# Supplementary material for: Smart textile lighting/display system with multifunctional fibre devices for large scale smart home and IoT applications
Source: Nat Commun. 2022 Feb 10;13:814. doi: 10.1038/s41467-022-28459-6 (PMC8831553; doi:10.1038/s41467-022-28459-6)
Supplement: Supplementary file 3 — Description of Additional Supplementary Files [file 41467_2022_28459_MOESM3_ESM.pdf]

Description of Additional Supplementary Files

File Name: Supplementary Movie 1
Description: Display Mode

File Name: Supplementary Movie 2
Description: Mechanical Test

File Name: Supplementary Movie 3
Description: F-RF antenna

File Name: Supplementary Movie 4
Description: F-photodetector

File Name: Supplementary Movie 5
Description: F-temperature sensor

File Name: Supplementary Movie 6
Description: F-biosensor module

File Name: Supplementary Movie 7
Description: F-touch sensor

File Name: Supplementary Movie 8
Description: F-energy storage

File Name: Supplementary Movie 9
Description: IPX7 Water resistance test
